# Select Small Non‐Coding RNAs Are Determinants of Survival in Older Adults

**DOI:** 10.1111/acel.70403

**Published:** 2026-02-24

**Authors:** Virginia Byers Kraus, Sisi Ma, Syeda Iffat Naz, Xin Zhang, Christopher G. Vann, Melissa C. Orenduff, William E. Kraus, Steven Shen, Janet L. Huebner, Ching‐Heng Chou, Erich Kummerfeld, Harvey Jay Cohen, Constantin F. Aliferis

**Affiliations:** ^1^ Duke Molecular Physiology Institute, and Duke Department of Medicine Duke University Durham North Carolina USA; ^2^ Institute for Health Informatics, and University of Minnesota Department of Medicine University of Minnesota Minneapolis Minnesota USA; ^3^ Institute for Health Informatics University of Minnesota Minneapolis Minnesota USA; ^4^ Duke Molecular Physiology Institute, and Duke Department of Orthopaedic Surgery Duke University Durham North Carolina USA; ^5^ Duke Molecular Physiology Institute Duke University Durham North Carolina USA; ^6^ Duke Molecular Physiology Institute, Duke Department of Medicine Duke University Durham North Carolina USA; ^7^ Center for the Study of Aging and Human Development Duke University Durham North Carolina USA; ^8^ Institute for Health Informatics, University of Minnesota Clinical and Translational Science Institute, and University of Minnesota Department of Medicine Minneapolis Minnesota USA

**Keywords:** aging, causality, epigenetic processes, HDL lipoproteins, microRNAs, piwi‐interacting RNA, RNA, survival

## Abstract

To investigate the relevance of small RNAs to human longevity, we pursued three goals: (a) to validate epigenetic (small RNA) factors underlying survival of older adults, (b) to develop and validate prediction models of survival for potential clinical application, and (c) to identify plausible druggable targets prolonging longevity. We evaluated 828 small non‐coding RNAs—687 microRNAs (miRNAs) and 141 piwi‐interacting RNAs (piRNAs)—in baseline plasma from 1271 community‐dwelling older adults (≥ 71 years) in the Duke‐EPESE study. Our predictive model incorporating smRNAs, clinical variables (demographics, lifestyle, mood, physical function, standard clinical laboratory tests, NMR‐derived lipids and metabolites, and medical conditions) and age achieved strong performance, with cross‐validated AUCs of 0.92 for 2‐year survival in Discovery and 0.87 in external Validation. Nine piRNAs, all reduced in longer‐lived individuals, were identified as potential therapeutic targets. Under the assumption of causal sufficiency, these data provide causal evidence linking circulating small RNAs with survival outcomes in humans. While such inference does not replace experimental validation, it complements mechanistic studies by identifying candidate molecular drivers most relevant to human longevity. Supporting biological plausibility, reduced piRNA biogenesis has been shown to double lifespan in C elegans. Together, our findings identify circulating piRNAs and miRNAs as promising biomarkers and potential therapeutic targets to advance human longevity.

## Graphical abstract

Circulating small RNAs were profiled by RNA sequencing in a well‐characterized cohort and evaluated alongside 187 clinical variables to predict survival. Models incorporating circulating small RNAs, including parsimonious subsets, outperformed age and clinical factors alone (AUC), with causal analyses supported by experimental evidence from the literature.

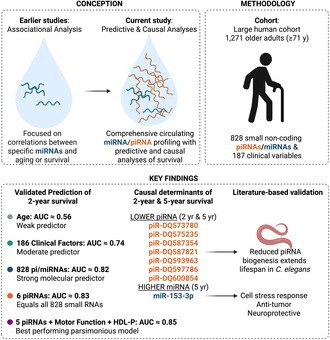

Small non‐coding RNAs (smRNAs), including PIWI‐interacting RNAs (piRNAs) and microRNAs (miRNAs), are emerging as important regulators in various biological processes related to aging, such as FOXO/DAF‐16, insulin/IGF signaling, and stress response mechanisms like HSF‐1 and Nrf/SKN‐1, which modulate longevity across species (Grillari and Grillari‐Voglauer 2010; Kim and Lee 2019; Simon et al. 2014). The roles of miRNAs in human aging and longevity have been explored (Eshkoor et al. 2022; Wang et al. 2024, 2023), with circulating miRNAs showing promise as lifespan‐modulating biomarkers (Kinser and Pincus 2020), highlighting their potential as non‐invasive indicators for aging‐related studies, while the roles of piRNAs remain under‐investigated, especially in humans.

PiRNAs are traditionally considered components of the innate immune defense system in the germline (Grillari and Grillari‐Voglauer 2010), where they help maintain genome stability by silencing transposons through cytosine methylation. Although they represent the largest class of small non‐coding RNAs in animal cells (Jiang et al. 2024), and have been detected in somatic cells (Balaratnam et al. 2018; Lin and Yin 2008; Malone et al. 2009; Mei et al. 2015), their functions outside the germline remain poorly understood. In humans, transposon derepression has been shown to stimulate anti‐tumor T cell immunity (Sheng et al. 2018) and regulate host gene expression during viral infections (Shen et al. 2022); however, the relationship between circulating piRNAs and human longevity remains largely unexplored. Studies in model organisms offer important clues (Janic et al. 2010; Jones et al. 2016; Proshkina et al. 2021; Shi and Murphy 2023; M. Simon et al. 2014; Zhu et al. 2020). In *C. elegans*, disruption of piRNA biogenesis components—e.g., via TOFU‐1 (Goh et al. 2014) knockdown—can significantly extend (double) organismal lifespan (Huang et al. 2023). In *Drosophila*, tissue specific knockdowns of *piwi* (a member of the Argonaute protein family primarily known for binding piRNAs) in the nervous system and fat body significantly alters lifespan in a manner dependent on sex, tissue type and stress state (Proshkina et al. 2021).

These results do not guarantee translation to humans. We therefore aimed to achieve three overarching goals: (a) to identify and validate smRNAs as epigenetic factors—regulators that modulate gene expression without altering the underlying DNA sequence (Jouravleva and Zamore 2025)—implicated in human longevity; (b) to develop and validate prediction models of survival for eventual clinical use; and (c) to identify plausible smRNA drug targets for prolonging survival. We hypothesize that smRNAs are key determinants of survival in humans. To achieve these goals, we conducted a non‐targeted analysis of circulating smRNAs, focusing on piRNAs in addition to miRNAs, to identify epigenetic molecular mechanisms causally linked to survival, and to identify potentially novel biomarkers of survival and healthy aging.

Achieving these goals and testing this hypothesis required integrating predictive and causal modeling approaches. We used specialized algorithms (Statnikov et al. 2013) capable of identifying all Markov Boundaries (MBs) of survival to simultaneously capture both maximally predictive and causally determinant factors of the complex biological outcome of survival. MB analysis, rooted in Pearl and Spirtes et al. causal graph theory (Pearl 2009; Spirtes et al. 2001), and extended—in terms of biomarker selection, scaling to large dimensionalities, equivalence class modeling, and algorithm‐guided experimental validation—for biomedical applications by Aliferis et al. (Aliferis et al. 2010a, 2010b; Statnikov and Aliferis 2010; Statnikov et al. 2013; Statnikov et al. 2015), is particularly well‐suited for identifying potential proximal molecular and lifestyle intervention targets. This method ensures predictive reliability while uncovering complex causality from observational or mixed experimental/observational datasets, potentially reducing the need for large‐scale follow‐up experiments for validation (Aliferis and Simon 2024b). By applying these methods to analyze circulating smRNAs, particularly piRNAs, we aimed to elucidate their functional roles in somatic cell biology and their broader contributions to the biological processes underlying aging, survival, and longevity.

## 1 Results

### 1.1 EPESE Cohort

Our analyses are based on 1271 participants from the community‐based Duke‐Established Populations for Epidemiologic Studies of the Elderly (D‐EPESE) cohort, all aged ≥ 71 years at the time of blood sample collection in year six of the study (1992–93). In brief, D‐EPESE was a multi‐year study sponsored by the National Institute on Aging to describe and identify predictors of mortality and investigate risk factors for chronic diseases and loss of functioning (Blazer and George 2012). The North Carolina cohort, established in 1986, was a sample of persons 65 years or older residing in households in five counties in the Central Piedmont area. This unique NC cohort, with over 50% Black participants at baseline and drawn from a region encompassing diverse racial and urban–rural populations, enabled comparisons of risk factors and disease distribution across these groups.

### 1.2 Biomarker Discovery and Internal Validation

**FIGURE 1:**
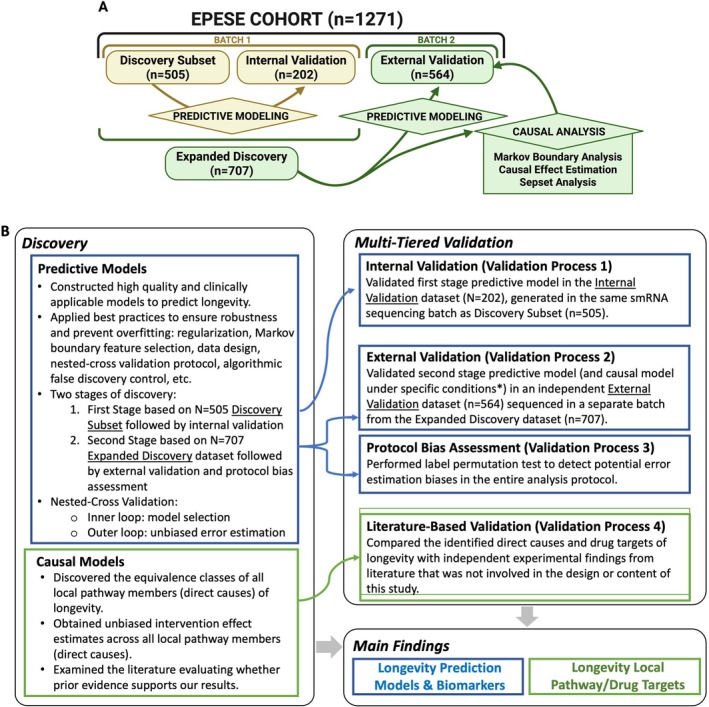
Analytical study design. (A) Leveraging molecular and clinical data from 1271 participants in the D‐EPESE study, we built models to identify predictors and causes of survival at different time horizons (2‐, 5‐, and 10‐years) following best practices for complex statistical machine learning modeling to ensure unbiased and generalizable estimation of predictive performance and causal relevance. The Expanded Discovery dataset (*N* = 707; smRNA sequenced in one batch) was split into a Discovery Subset (*N* = 505) and an Internal Validation set (*N* = 202), while an independent External Validation set (*N* = 564), drawn from a separately withheld half of the D‐EPESE cohort and sequenced in a different batch, was used to evaluate model performance, generalizability, and robustness to smRNA measurement variability. (B) Summary of the analytical design. Symbols in blue denote predictive modeling analyses and in green denote causal modeling analyses. *Predictive Modeling* was conducted to build and validate predictive models for longevity based on smRNA with and without other patient characteristics. For the first stage model discovery and internal validation, we aimed to establish internal validity of our models given smRNA data measured within the same assay batch. We used the Discovery Subset to build predictive models and conduct model selection (inner loop of the stratified repeated nested cross‐validation [NCV]). For the second stage of model discovery, we aimed to establish external validity of our models, specifically, the generalizability of our models across different (non‐overlapping) batches of biological samples and assays. We used the Expanded Discovery data for model selection and performance estimation via NCV and built the final predictive model (with the best hyperparameters identified), and then subsequently and independently tested the model on independent External Validation data (*N* = 564) where smRNAs were sequenced as a separate batch. A label permutation test was conducted to assess potential error estimation biases. *Causal Modeling* was conducted to identify potential proximal causes (local pathways) for survival using the Expanded Discovery data with Markov Boundary analysis. The causal effects of the identified causes were also estimated. Sepset analyses were conducted to gain more biological insights into the causal role of the study variables. These results were cross‐checked with prior findings from the literature. Panel A created in BioRender, agreement FD287GWPQ8.

To discover and validate plasma smRNAs (piRNAs and miRNAs) with predictive and causal relevance for survival, we employed a rigorous, multi‐stage protocol to ensure unbiased model selection, error estimation, and generalizability (Figure 1). We used next‐generation sequencing to quantify smRNA levels in 707 participants from the D‐EPESE cohort (Kraus et al. 2022). This “Expanded Discovery” dataset comprised a random half of the D‐EPESE cohort with available plasma biospecimens and, was originally selected using stratified randomization to ensure balance by race, sex, age, and survival status (Methods S1). In the first stage of discovery and validation, the Expanded Discovery dataset was further randomly divided into a “Discovery Subset” dataset (*n* = 505) and an “Internal Validation” dataset (*n* = 202) (Figure 1). All data splits preserved the proportion of surviving and non‐surviving participants across both the inner and outer loops (see Section 3). Using the Discovery Subset dataset, we built predictive models incorporating 828 smRNAs, age, or 186 clinical features, and their combinations, to predict survival across 2‐, 5‐, and 10‐year intervals following the timepoint of plasma acquisition used for profiling smRNA. The 186 clinical features consisted of sociodemographic variables including demographics and anthropometric measures (age, race, sex, education, income, body mass index [BMI]), medical morbidities, self‐rated health, depression, health behaviors (smoking habits, alcohol use, sleep), cognitive and physical/motor activity status, and soluble analytes including 48 traditional medical blood tests, 6 additional inflammation parameters, and 48 lipoprotein biomarkers (NMR LipoProfile blood test) (Kraus et al. 2022).

Models were trained on the Discovery Subset dataset (*n* = 505), with unbiased performance estimates obtained from the outer loop of nested cross‐validation (NCV) and from the Internal Validation dataset (*n* = 202). The smRNA‐only model for 2‐year survival achieved cross‐validated AUC 0.89 ± 0.02 in the Discovery Subset and validation AUC 0.91 (95% CI: 0.84–0.98) in the independent Internal Validation dataset (Table S1), demonstrating strong predictive performance of smRNAs for 2‐year survival in the Expanded Discovery data overall. The smRNA‐only models demonstrated lower accuracy for 5‐ and 10‐year predictions, yielding AUCs 0.63 and 0.65, respectively, in the independent Internal Validation dataset (Table S1). Models incorporating smRNAs, age, and clinical features improved predictive accuracy for 5‐year survival (AUC 0.77) but showed limited enhancement of 10‐year predictions (AUC 0.66). These findings highlight the strong short‐term predictive value of smRNAs while emphasizing the need for integrated models to improve survival predictions over extended timeframes. Additional predictive performance details for other models and combinations can be found in Table S1.

As part of the discovery process—and to simplify clinical testing for optimal quality and consistency—we compared the performance of the initial Qiagen platform‐specific output (smRNA_PSO_) with data normalized using the standard miRNA normalization method, smRNA_TMM_ (normalized Trimmed Mean of M‐values [TMM values]) (Robinson and Oshlack 2010). Across both the Discovery Subset and Internal Validation datasets, smRNA_PSO_ and smRNA_TMM_ demonstrated statistically comparable predictive performance (*p* > 0.05) for most outcomes (Table S2). Given its simplicity and greater practicality for clinical use, we selected smRNA_PSO_ for all subsequent analyses to streamline future clinical applications.

### 1.3 External Validation in an Independent Cohort With Separate smRNA Sequencing

To strengthen bias prevention and to assess generalizability associated with sequencing batch effects, a double‐holdout design was instituted (Figure 1). In addition to the nested cross validation followed by internal validation described above, an external validation stage was implemented. To create a separate group of individuals, distinct from the data used to build the model to assess the model's performance, generalizability, and robustness to variability in smRNA measurements, we used an independent sample of 564 participants from D‐EPESE. These individuals were drawn from the remaining randomly withheld half of the D‐EPESE cohort; the plasmas of the independent External Validation cohort were processed and sequenced for smRNA in a separate batch, using the same methods as for the Expanded Discovery dataset but with a slightly greater depth of sequencing (see Section 3). Descriptive statistics in Table 1 present clinical features for each D‐EPESE sample dataset; descriptive statistics in Table S3 present clinical features and smRNAs of Markov Boundaries (MBs, i.e., from causal analysis) for the Expanded Discovery dataset by survival status (alive/dead) for each time horizon. Methods followed established best practices (Aliferis and Simon 2024a, 2024c) (see Section 3 and Methods S1), including an outcome label permutation test (Ojala and Garriga 2010) (Figure 1, Table S4) to confirm unbiasedness. This comprehensive protocol—integrating model selection and fitting, error estimation, bias avoidance, and causal interpretation—validated smRNAs as robust and clinically useful biomarkers of survival. Results are described below for the Expanded Discovery cohort (*n* = 707) and the independent External Validation cohort (*n* = 564).

**TABLE 1:** Characteristics of D‐EPESE cohort, sample subsets and clinical features selected in Markov Boundary (MB) analyses.

Dataset	Discovery Subset dataset, *N* = 505	Internal Validation dataset, *N* = 202	Expanded Discovery dataset (combined Discovery Subset and Internal Validation dataset), *N* = 707	Independent External Validation dataset, *N* = 564	Time Horizon for which a feature was selected as part of the MBs (year)
	Mean (SD)	Mean (SD)	Mean (SD)	Mean (SD)	
Age at baseline (years)	77.98 (5.34)	78.12 (5.57)	78.02 (5.40)	78.42 (5.54)	5
Sex (% Female)	65.15	67.33	65.77	63.48	NA
Body Mass Index (P3) (kg/m^2^)	25.95 (4.62)	25.76 (5.31)	25.89 (4.82)	25.90 (5.02)	NA
Change in Body Mass Index (P2 to P3)	−0.16 (2.32)	−0.23 (2.50)	−0.18 (2.37)	−0.52 (2.86)	5
Total HDL particles	17.86 (3.31)	17.94 (3.31)	17.88 (3.31)	17.88 (3.42)	2, 5
Small HDL particles (< 9 nm)	13.38 (3.36)	13.67 (3.13)	13.46 (3.30)	13.33 (3.25)	2, 5
GlycA (μmol/L)	422.89 (79.46)	422.61 (74.45)	422.81 (78.00)	424.57 (84.25)	5
Alpha‐2 Globulins (g/dL)	0.73 (0.18)	0.72 (0.18)	0.73 (0.18)	0.73 (0.17)	5
Blood Urea Nitrogen (mg/dL)	18.89 (7.56)	19.66 (9.08)	19.11 (8.03)	19.44 (9.76)	5
Creatinine (mg/dL)	1.34 (0.43)	1.34 (0.49)	1.34 (0.45)	1.36 (0.78)	5
Carbon Dioxide (milli‐equivalents/L)	28.57 (4.32)	28.50 (4.36)	28.55 (4.33)	28.09 (4.49)	5, 10
Selectin (pg/mL)	459.49 (214.67)	456.13 (162.94)	458.54 (201.24)	494.85 (779.17)	5
Valine (μmol/L)	218.36 (49.67)	215.35 (50.22)	217.49 (49.81)	215.59 (49.92)	10
Neutrophils (%)	59.78 (9.83)	59.19 (10.93)	59.61 (10.16)	59.88 (9.24)	5
Lymphocytes (%)	31.43 (9.27)	32.09 (10.11)	31.62 (9.52)	31.20 (8.79)	5
Lymphocytes (absolute count of number × 10E3 per cubic mm)	2.01 (1.07)	2.00 (0.67)	2.01 (0.97)	1.97 (1.02)	5
IADL‐motor (higher better)	3.25 (1.29)	3.18 (1.27)	3.23 (1.29)	3.22 (1.30)	2, 5, 10
IADL‐cognitive (higher better)	2.67 (0.76)	2.69 (0.70)	2.67 (0.74)	2.68 (0.71)	5
Cognitive Impairment (0 no impairment, 1 impairment)	0.12 (0.33)	0.14 (0.34)	0.13 (0.33)	0.12 (0.33)	5, 10
Rosow‐Breslau Score: Able to walk ½ mile (higher score able to do)	1.68 (0.47)	1.64 (0.48)	1.67 (0.47)	1.63 (0.48)	5
Help Bathing^a^ (lower better)	1.10 (0.30)	1.09 (0.29)	1.10 (0.30)	1.10 (0.31)	5
Help Toileting^a^ (lower better)	1.07 (0.28)	1.07 (0.25)	1.07 (0.27)	1.09 (0.30)	5
Total Rosow‐Breslau Score Sum of 3 items (higher score more impaired)	0.97 (1.15)	1.02 (1.17)	0.98 (1.16)	1.05 (1.19)	5
Health Index Score (continuous, lower better)	45.15 (34.76)	42.98 (32.70)	44.53 (34.18)	47.88 (35.39)	5
Health Index Score (trichotomized, lower better)	1.24 (0.73)	1.20 (0.75)	1.23 (0.74)	1.27 (0.75)	5
How often do you walk a mile or more without resting? (lower better)	5.07 (1.60)	5.23 (1.45)	5.11 (1.56)	5.04 (1.72)	10

*Note:* Except where indicated, all measures were obtained at P3 (third in‐person visit with blood acquisition in 1992); P2 in‐person visit 1989; SD = standard deviation; IADL‐motor = # motor function‐related Instrumental Activities of Daily Living (IADL) items CAN do (traveling, shopping, preparing meals, doing housework); IADL‐cognitive = # cognitive function‐related Instrumental Activities of Daily Living (IADL) items CAN do (telephone use, taking medications, managing finances).

Abbreviations: HDL, high density lipoprotein; NA, not applicable as the feature was not selected in any model.

^a^ 1 = need no help, 2 = need help, 3 = unable to do.

#### 1.3.1 Predictive Model Performance in 2‐, 5‐, and 10‐Year Survival Horizons

For 2‐year survival (Table 2 and Table S4 with full details), the predictive model built with smRNA_PSO_ data achieved nested cross‐validated AUC 0.90 ± 0.01 in the Expanded Discovery dataset (*n* = 707) and AUC 0.82 (0.78, 0.87) in the independent External Validation dataset (*n* = 564) that underwent smRNA sequencing in a separate batch. The predictive performance of smRNA_PSO_ was significantly better (*p* < 0.05) than models built with age or clinical variables alone: age cross‐validated AUC 0.57 ± 0.03 and External Validation AUC 0.56 (0.50, 0.62); and clinical variables cross‐validated AUC 0.79 ± 0.02 and External Validation AUC 0.74 (0.69, 0.80). Compared with models using smRNA_PSO_ alone, combining smRNA_PSO_, age, and clinical variables resulted in improved predictive performance in the cross‐validated Discovery (AUC 0.92 ± 0.01, *p* = 0.03), but comparable performance in the External Validation dataset (AUC 0.87 [0.83, 0.90], *p* = 0.35).

**TABLE 2:** Performance of 2‐year survival models with and without optimization for clinical deployment.

	Predictive models					Streamlined predictive models for clinical deployment				
	NCV AUC	External validation AUC	# Features	Feature selector	Classifier	NCV AUC	External validation AUC	# Features	Feature selector	Classifier
	Mean (SD)	Value (95% CI)				Mean (SD)	Value (95% CI)			
smRNA	0.90 (0.01)	0.82 (0.78, 0.87)	828 smRNA	All features	Random Forest	0.86 (0.01)	0.83 (0.78, 0.87)	6 piRNA^a^	GLL‐K‐3	GLM
Age	0.57 (0.03)	0.56 (0.50, 0.62)	1 age	All features	GLM	0.58 (0.02)	0.56 (0.49, 0.62)	1 age	All features	GLM
Clinical	0.79 (0.02)	0.74 (0.69, 0.80)	186 clinical	All features	Random Forest	0.76 (0.01)	0.73 (0.68, 0.79)	7 clinical^b^	GLL‐K‐3	GLM
smRNA +Age +Clinical	0.92 (0.01)	0.87 (0.83, 0.90)	828 smRNA +age +186 clinical	All features	Random Forest	0.90 (0.02)	0.85 (0.80, 0.89)	5 piRNA + 2 clinical^c^	GLL‐K‐3	GLM

*Note:* Performances were estimated in the outer loop of the nested cross validation (NCV) of the Expanded Discovery (*n* = 707) data (NCV AUC) and independently on the External Validation (*N* = 564) data (External Validation AUC).

Abbreviations: GLL‐K‐3, Generalized local learning with max‐k = 3; GLM, generalized linear model, specifically logistic regression.

^a^ The six piRNAs are PIR‐ DQ573780, DQ587552, DQ587821, DQ593963, DQ597786, DQ575235.

^b^ The seven clinical factors are total high density lipoprotein particle number (Total HDL‐P), instrumental activities of daily living related to physical function (IADL‐motor), albumin, health index score (continuous measure at P3 time of blood draw), gamma‐glutamyl transferase (GGT), monocytes (absolute count), pack years of cigarette smoking (self‐reported at T2, the second telephone interview).

^c^ The 5 smRNA+2 clinical variables are PIR‐ DQ573780, DQ587821, DQ593963, DQ597786, DQ57523, IADL‐MOTOR, and Total HDL‐P.

For 5‐year survival (Table S4), most models showed lower predictive performance than for 2‐year survival. Models with smRNA_PSO_, age, or clinical variables achieved independent External Validation AUCs 0.61 (0.57, 0.66), 0.64 (0.59, 0.68), and 0.74 (0.68, 0.77), respectively. Compared to models using clinical variables alone, combining smRNA_PSO_, age, and clinical variables did not improve the predictive performance in the External Validation dataset (AUC 0.75 [0.71, 0.79], *p* = 0.73). For 10‐year survival (Table S4), the predictive performance of smRNAs, age, clinical variables, and their combinations was even weaker, with cross‐validated Discovery AUCs < 0.7 and External Validation AUCs ≤ 0.65 across all models. In short, as the prediction time horizon increased (i.e., as the time from blood acquisition to survival outcome lengthened), the predictive performance declined.

#### 1.3.2 Streamlined Predictive Models for Practical Clinical Deployment

Many of the highest‐performing models included multiple variables. For example, the top 2‐year survival model (cross‐validated Discovery AUC 0.92 ± 0.01; External Validation AUC 0.87 [0.83, 0.90]) used all 828 smRNA_PSO_, all 186 clinical variables, and age. To improve clinical applicability, we next sought to develop simpler, more parsimonious models that used fewer variables without compromising predictive accuracy. We therefore restricted model inputs to MB features, which theoretically achieve maximum simplicity while retaining all non‐redundant predictive information. The MBs were identified using the generalized local learning (GLL) algorithm family (Aliferis et al. 2010a, 2010b) and the TIE* algorithm (Statnikov et al. 2013) (see Methods S1). Using this streamlined approach, we developed a 2‐year survival model containing only six piRNAs (piR‐DQ573780, piR‐DQ587552, piR‐DQ587821, piR‐DQ593963, piR‐DQ597786, piR‐DQ575235). This reduced model preserved nearly all the predictive power of the full 828 smRNA model (Discovery AUC 0.86 ± 0.02 vs. 0.90 ± 0.01, *p* < 0.01; External Validation AUC 0.83 [0.78, 0.87] vs. 0.82 [0.78, 0.87], *p* > 0.05). When age and clinical variables were incorporated, a parsimonious model containing just 5 piRNAs (piR‐DQ573780, piR‐DQ587821, piR‐DQ593963, piR‐DQ597786, piR‐DQ575235) and 2 clinical variables—instrumental activities of daily living related to physical function (IADL‐motor) and total high density lipoprotein particle number (Total HDL‐P)—maintained performance comparable to the full model with all 828 smRNAs, age, and all 186 clinical variables (Table S4). The simplified model achieved a Discovery AUC 0.90 ± 0.02 vs. 0.92 ± 0.01 for the full model (*p* < 0.03) and an External Validation AUC 0.85 [0.80, 0.89] vs. 0.87 [0.81, 0.90] (*p* > 0.05). Across all time horizons, streamlined predictive models yielded performance metrics comparable to their larger counterparts (Table 2 and Table S4). Label permutation tests confirmed that our predictive modeling protocol was unbiased and statistically tested, produced signatures against the null hypothesis of no signal (Table S4).

### 1.4 Algorithmic Discovery of Markov Boundaries and Potential Direct Causes of Survival Across Time Horizons

#### 1.4.1 Identification of smRNAs and Clinical Variables That Are Potential Direct (Proximal) Causes of 2‐Year Survival

**FIGURE 2:**
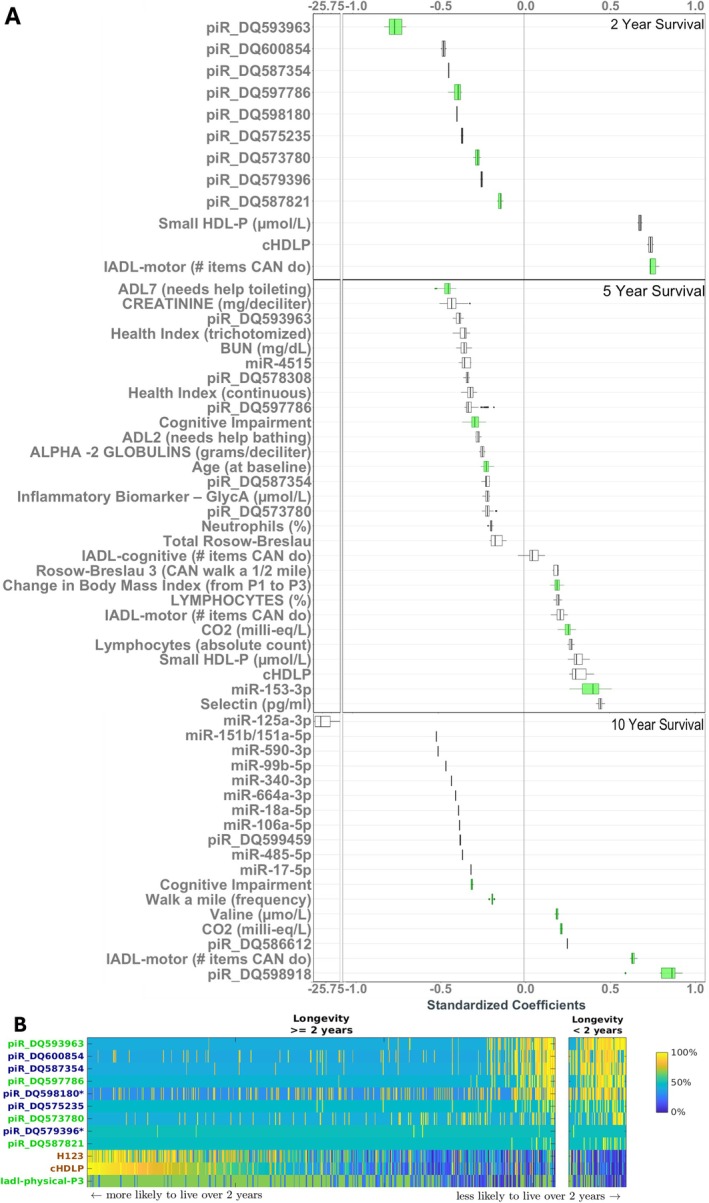
Markov Boundaries of longevity. (A) The most critical predictors of longevity across different time horizons, called Markov Boundary (MB) variables, were identified by machine learning. The MB variables are also potential direct causes given that longevity is a terminal variable under causal sufficiency. We estimated their ranges of estimated effect sizes for predicting 2‐year (top panel), 5‐year (middle panel) and 10‐year (lower panel) longevity. All molecular variables were derived from measures at the time of the blood draw—the third in‐person evaluation (P3). Green indicates variables that appeared in all MBs. The total sample size *n* = 707. The box in the figure represents the range of the estimates for each variable from models with the variable present. The left (vertical) line of the box represents the 1st quantile (Q1), the right line of the box represents the 3rd quantile (Q3), the internal line represents the median, the left whisker = min(max(*x*), Q1 + 1.5 × IQR), the right whisker = max(min(*x*), Q3 – 1.5 × IQR), where IQR is the interquartile range. The large coefficient of miR‐125a‐3p is a result of its absence in all participants who survived beyond 10 years. Results are generated using smRNA_PSO_, that is, the platform specific outputs. (B) Heatmap illustrating the empirical distribution of the MB variables (rows) of 2‐year longevity over all participants (columns), stratified by longevity ≥ or < 2 years. Variables are ranked by effect sizes from negative to positive. Values of the variables were converted to percentiles for illustration purposes. Percentile numbers were represented with different colors (see color bar on the right). Variables names marked in green appears in all MBs. The variables with names marked in red contain the same information about 2‐year longevity given other variables in the MB. The two variables marked with * contain the same information as all other variables that are marked in blue. IADL‐physical‐P3 is instrumental activities of daily living motor function at the in‐person visit of the blood draw.

The previous analysis identified MBs of survival at different time horizons. By MB theory, MBs of survival comprise the local causal pathway of survival in distributions without target information equivalence. In omics distributions, significant information equivalencies among molecules for a target phenotype (e.g., survival) often exist, where optimal and statistically equivalent information about the phenotype can be obtained from multiple sets of variables. Ignoring target information equivalence can result in false positive and false negative discovery of causal determinants (Kraus et al. 2022; Simon and Aliferis 2024; Statnikov et al. 2013). To capture all statistically equivalent putative causes, we conducted MB discovery on all 1015 variables representing the combined set of smRNAs, age, and clinical features (Figure 2), and from smRNA data alone (Figure S1). From the combined set of all data, the members of the MBs identified for predicting 2‐year survival included nine piRNAs (no miRNAs), small HDL (H123) particle number, total HDL particle concentration (cHDLP), and IADL‐motor function (Table S3). Each MB consisted of 7–8 variables, achieving External Validation AUC 0.84 (SD 0.01) (Table S5). These variables are *potential* direct causes of 2‐year survival. Of these 9 selected piRNAs, four piRNAs (piR‐DQ573780, piR‐DQ587821, piR‐DQ593963, piR‐DQ597786), IADL‐motor, and HDL particle concentration (total or small) appeared in 100% of MBs (Figure 2, Table S3); given causal sufficiency (i.e., lack of unmeasured causal confounding), these variables in the intersection of all MBs identified by the algorithm are expected to be direct causes of 2‐year survival. Of the nine piRNAs selected as predictors of 2‐year survival, four piRNAs (DQ573780, DQ587354, DQ593963, and DQ597786) not in the intersection of all MBs, were also *potential* direct causes of 5‐year survival, with the remainder of the direct causes guaranteed to be in this set plus false positives. All nine piRNAs were consistently lower in the long‐lived compared to short‐lived individuals (Table S3); this was an a priori unexpected finding, given the established role of piRNAs in suppressing transposon activity in the germline (Siomi et al. 2011) and the double‐stranded DNA breaks generated by transposition, that are believed to serve as a mechanism to regulate the pace of aging (Lenart et al. 2018).

As we previously observed (Kraus et al. 2022), better motor function (IADL‐motor) and higher HDL particle (total and small) counts were estimated to positively impact survival, appearing in all MBs. The strongest predictors of 2‐year survival were piR‐DQ593963 and IADL‐motor, with average standardized effect estimates (sES) in the logistic regression model (see details on causal effect estimation in Section 3) of −0.752 and +0.754, respectively (Figure 2, Table S6a). Notably, among the measured variables, age at study baseline did not emerge as a direct cause of 2‐year survival after blood acquisition (8 years after baseline). This suggests that once the relevant molecular factors are identified, age—primarily serving as a proxy for these factors—did not independently influence short‐term survival (see the Section 1.4.4 for further details).

Since smRNAs provide an aging biomarker that is potentially modifiable, we estimated the degree of improvement in survival by a theoretical (in silico) “intervention” consisting of adjusting the values of the smRNA variables identified as potential direct causes of survival for each participant to that of the 5th percentile (if the variable was estimated to hamper survival) or 95th percentile (if the variable was estimated to promote survival) (Table S7, right panel). The estimated effect of this “intervention” was improved probability of 2‐year survival from 47% to 90% in the Expanded Discovery dataset and from 73% to 94% in the External Validation dataset. In relative terms, these represent approximately 1.9‐fold and 1.3‐fold increases in survival probability. Expressed inversely, the corresponding 2‐year probability of death declined from 53% to 10% (a 5.3‐fold reduction) in the Expanded Discovery dataset and from 27% to 6% (a 4.5‐fold reduction) in the External Validation dataset.

When all potential direct causes were jointly modified—representing a hypothetical combined lifestyle and pharmaceutical intervention—the probability of 2‐year survival increased from 47% to 99.6% in the Expanded Discovery dataset and from 73% to 99.6% in the External Validation dataset (2.1‐fold and 1.4‐fold increases in survival, respectively). Correspondingly, the probability of death was reduced from 53% to 0.4% (a 133‐fold reduction) in the Expanded Discovery dataset and from 27% to 0.4% (a 68‐fold reduction) in the External Validation dataset (Table S7, left panel). These results underscore the key role of these causal factors—particularly smRNAs—in 2‐year survival.

#### 1.4.2 Identification of smRNAs and Clinical Variables That Are Potential Direct (Proximal) Causes of 5‐ and 10‐Year Survival Horizons

The set of equivalent MBs predicting 5‐year survival consisted of 115 MBs. Each MB contained 13–15 variables, achieving AUC 0.73 ± 0.01 in the independent External Validation dataset (Table S5, Figure 2, and Table S6b). Seven smRNAs (2 miRNAs and 5 piRNAs) were identified as potential direct causes for 5‐year survival. These findings further support a distinct causal role of piRNAs and miRNAs in survival. Lesser need for toileting assistance, higher miR‐153‐3p and serum CO_2_ levels, modest BMI increase, and absence of cognitive impairment were consistently associated with greater 5‐year survival, while age remained a key but indirect proxy for unmeasured or time‐varying molecular factors. A model‐based “intervention” on MB smRNAs alone in the independent External Validation cohort corresponded to an increase in the estimated 5‐year survival probability among non‐survivors from 61% to 73% (Table S7), corresponding to a 1.2‐fold improvement in survival. Expressed inversely, the corresponding probability of death declined from 39% to 27%, representing a 1.4‐fold reduction in 5‐year mortality.

Ten MBs predicted 10‐year survival equally well though with lower predictive performance compared to models for 2‐ and 5‐year survival. Each MB consisted of 7–9 variables, achieving AUC 0.61 ± 0.01 in the External Validation dataset (Table S5, Figure 2, and Table S6c). Both piRNAs (3 total) and miRNAs (10 total) were identified as potential causes, but none overlapped with those that predicted 2‐ and 5‐year survival. Physical function also remained a key potential cause (Table S6c). A model‐based “intervention” on MB smRNAs in the independent External Validation cohort increased the estimated 10‐year survival probability from 25% to 77% (Table S7), corresponding to a 3.1‐fold improvement in long‐term survival. The associated 10‐year probability of death declined from 75% to 23%, a 3.3‐fold reduction in mortality.

#### 1.4.3 Identification of Causal Predictive smRNAs for Survival Independent of Clinical Variables

To align with clinical practice, which often relies on blood‐based biomarkers, we generated MBs using smRNA data alone, excluding clinical data and age. For predicting 2‐year survival, smRNA‐derived MBs were highly robust. Three MBs were discovered, wherein each MB contained 6 smRNAs for a total of eight smRNAs overall; all MB smRNAs were piRNAs—seven negatively and one positively associated with 2‐year survival. These MBs achieved AUC 0.81 ± 0.02 on the External Validation dataset (Table S5, Table S6a right panel, Figure S1). The predictive performance of the smRNA‐derived MBs declined considerably for the longer time horizons compared to the combination of smRNAs, clinical variables, and age. For 5‐year survival, 18 MBs were identified consisting of 22 smRNAs (5 of which were piRNAs) yielding External Validation AUC 0.56 ± 0.04; for 10‐year survival, 3 MBs were identified consisting of 5 smRNAs (one of which was a piRNA) yielding External Validation AUC 0.55 ± SD 0.01 (Tables S5 and S6b,c right panels, Figure S1). These findings underscore the strong short‐term predictive power of piRNAs and suggest that additional factors are required for accurate long‐term survival predictions.

#### 1.4.4 Sepset Analyses

A “sepset” is a set of variables that causally mediates (or confounds) the effect (or association, respectively) of another variable on the outcome (survival), leading to the latter's exclusion from the MBs. Sepset analysis describes why particular variables are not selected in the MBs for survival, and therefore, are not considered potential direct causal factors for survival, even if they are correlated with it. Sepset analysis can help bring clarity to results within a study and across studies. For example, the total number of HDL particles was identified as a sepset for age, indicating that HDL particle levels encapsulated all 2‐year survival‐related information attributable to age (Table S8). Similarly, piR‐DQ593963 acted as a sepset for sex, while IADL‐motor (Fillenbaum 1988; Lawton and Brody 1969) was a sepset for other functional measures, such as Rosow‐Breslau mobility (Rosow and Breslau 1966), Nagi‐modified IADL and motor activity (Nagi 1976), and Katz basic activities of daily living (Katz et al. 1970). At the 2‐year time horizon, piR‐DQ593963 was the most frequently identified sepset.

At the 5‐year time horizon, miR‐153‐3p rendered most of the smRNAs irrelevant for 5‐year survival. piR‐DQ573780 and piR‐DQ593963 served as sepsets for the remaining smRNAs. Age was itself a sepset for several clinical factors, including serum protein, lipoprotein insulin resistance (LPIR), red blood cell count, hemoglobin, hematocrit, income, depression, and rarely feeling rested upon awakening (SLEEP5).

### 1.5 Identification of Putative smRNA Targets and Pathways

Because multiple databases use distinct naming conventions for piRNAs, Table S9 lists all identified piRNAs with their corresponding accession IDs, annotations across major databases (NCBI, piRNAQuest v2, piRBase, and piRNA_DB), genomic loci, sequence length, and nucleotide sequence and predicted gene targets; miRNAs are similarly annotated with their miRBase IDs and corresponding gene targets. Ten piRNAs predicting 2‐year survival targeted 65 mRNA transcripts, including four pseudogenes in addition to the Long Interspersed Nuclear Element‐1 (LINE‐1) retrotransposon (Table S9). The seven piRNAs predicting 5‐year survival targeted 19 mRNA transcripts, while the three piRNAs predicting 10‐year survival targeted eight mRNA transcripts and several miRNAs, notably the miR‐17‐92 cluster, with key roles in cell proliferation and survival, immune regulation, apoptosis, and oncogenesis (Grillari et al. 2010). No miRNAs were identified as predictors of 2‐year survival, while 18 miRNAs predicted 5‐year survival; among these, two were members of the miR‐17‐92 cluster and one a member of the miR‐106a‐363 paralogous cluster of the miR‐17‐92 cluster.

Using the interactive mSALT database (Tyshkovskiy et al. 2023)—a platform for visualizing associations between mammalian gene expression and aging or longevity traits—we expanded the biological understanding of four mRNA targets corresponding to three of the four smRNAs that appeared in 100% of MBs for this trait and were therefore identified as estimated direct causal drivers of 2‐year survival (Figure S2). Macro H2A.1 Histone (*MACRO2A1*, a potential target of piR‐DQ573780), a gene involved in chromosomal stability, transcriptional regulation, DNA repair and replication (Hernández‐Muñoz et al. 2005), is unchanged with aging in humans but consistently down regulated by longevity‐promoting interventions such as calorie restriction (CR), growth hormone deficiency, rapamycin, and other common pro‐longevity interventions. Potassium voltage‐gated channel interacting protein 4 (*KCNIP4*, also a potential target of piR‐DQ573780), which modulates neuronal excitability through regulation of potassium channels (Pruunsild and Timmusk 2005), shows a non‐significant decrease with age in humans. WEE2 antisense RNA 1 (*WEE2‐AS1*, a potential target of piR‐DQ593963) is a long non‐coding RNA (lncRNA) involved in meiotic cell division (Hanna et al. 2010), is reduced by CR and common pro‐longevity interventions. Solute carrier family 9‐member a7 (*SLC9A7*, a potential target of piR‐DQ597786), involved in regulation of cellular pH balance (Kagami et al. 2008), decreases with aging in humans and increases with CR. These expression patterns across species and interventions underscore the biological relevance of these smRNA targets in modulating lifespan.

**FIGURE 3:**
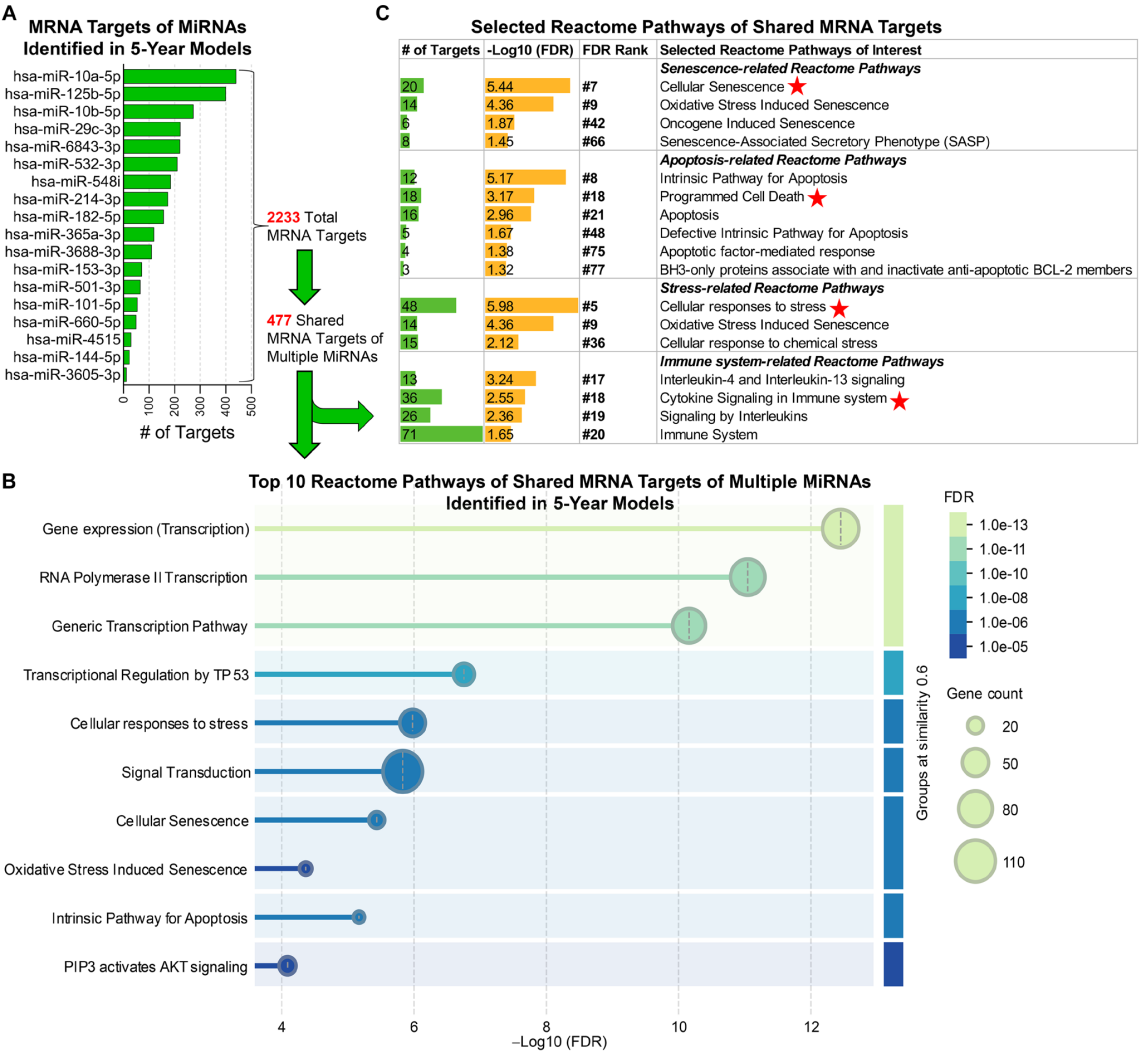
MiRNAs identified in 5‐year longevity–predictive models and their targets. (A) To identify mRNAs targets, the miRNAs that predicted 5‐year longevity were queried in MiRTarBase 9.0 and filtered for strong evidence, defined as direct experimental validation (e.g., reporter, Western blot, or qPCR assays). The bar graph displays the number of mRNA targets of the individual miRNAs, totaling 2233 mRNAs; among which 477 mRNAs were targets of multiple miRNAs. (B, C) Search Tool for the Retrieval of Interacting Genes/Proteins (STRING) network analyses were used to identify the significantly enriched (False Discovery Rate [FDR] < 0.05) Reactome Pathways of 474 targets; 3 targets (AHSA2, AZF1, and TSPEAR‐AS2) were not able to be mapped. The graphs display the top 10 Reactome Pathways ranked by FDR (B), and the selected Reactome Pathways related to senescence, apoptosis, stress and immune system (C). # in front of each pathway indicates the FDR rank among the total 79 identified Reactome Pathways.

Using Database for Annotation, Visualization, and Integrated Discovery (DAVID), we identified significant pathways through functional enrichment analysis of mRNAs predicted by survival‐associated smRNAs (Table S10); these included a longevity‐regulating pathway (Bonferroni *p* = 0.0001). The overall predicted targets were enriched for transcriptional, cell‐cycle, and stress/inflammatory signaling pathways—including MAPK, PI3K–Akt, TP53, and TLR cascades—indicating convergence on regulatory programs that govern proliferation, apoptosis, and cellular stress responses. Additional pathway analysis focused on the 5‐year survival predictors because this time horizon included a sufficient number of identified miRNAs and could be analyzed using available tools such as MiRTarBase 9.0 for mRNA target prediction. After filtering for “strong evidence”—defined by miRTarBase as interactions validated by direct experimental assays (e.g., reporter, Western blot, or qPCR; see Methods S1)—we identified 2233 mRNAs targeted by the 18 miRNAs, with each miRNA predicted to target 12–441 mRNAs. Notably, 477 mRNAs were targeted by multiple miRNAs (Figure 3A). STRING network analysis (Szklarczyk et al. 2019) revealed that these shared targets were highly interactive and enriched in 79 Reactome pathways. Four of the 79 pathways were senescence‐related; two of these, cellular senescence and oxidative stress‐induced senescence, were among the top 10 pathways (Figure 3B). Six pathways were apoptosis‐related, three stress‐response‐related, and four immune system‐related (Figure 3C). Additionally, many targets were implicated in regulating biosynthetic processes, mitochondrial function, metabolic processes and cell death (Figure S3).

## 2 Discussion

Yourman et al. (2012) identified six mortality prediction indices for community‐dwelling older adults, accessible through ePrognosis (Smith et al. 2011). These indices estimate 1‐ to 5‐year mortality risk based on 6–25 physician‐reported clinical factors—without incorporating laboratory‐based (molecular) measures—achieving a mean validation AUC of 0.74 (range 0.69–0.79). Using similarly accessible clinical data but including clinically accessible laboratory measures, we previously identified causal determinants of survival that achieved comparable predictive performance: 2‐year validation AUC of 0.74 (with 8 items) and 5‐year validation AUC of 0.77 (with 10–15 items) (Kraus et al. 2022). Building on this, we now show that using six piRNAs alone (External Validation AUC of 0.83), or five piRNAs combined with two clinical measures (External Validation AUC of 0.85), markedly improves prediction for 2‐year survival. The proposed 5‐piRNA plus 2‐clinical‐variable model is well suited for clinical translation. Notably, we determined that normalization of small RNA levels was unnecessary for model performance, simplifying assay implementation and reducing analytical complexity. Because our study was based on a community‐dwelling cohort, the model is expected to have reasonable generalizability to similar populations, though validation in broader and more diverse cohorts remains an important next step. The use of standard small RNA detection platforms (e.g., RT‐qPCR or targeted sequencing) would make clinical deployment feasible and cost‐efficient once the assay is standardized. Although smRNAs alone do not significantly enhance predictivity beyond clinically accessible factors for 5‐ and 10‐year survival, they offer insights into the molecular pathways of longevity, implicating cellular stress responses, senescence, apoptosis, and immune system regulation.

Epigenetic alterations are a primary hallmark of aging (López‐Otín et al. 2023). We observed that lower circulating levels of piRNAs were consistently associated with increased 2‐ and 5‐year survival. While piRNAs are traditionally associated with transposon silencing in germline cells, emerging evidence points to additional functions, including endonuclease, DNA methylation, modulation of histones, and m6A (RNA) methylation related to aging, antiviral defense, and cancer (Jiang et al. 2024; Siomi et al. 2011; Zhang et al. 2023). Of the 10 piRNAs associated with 2‐year survival in causal models, only two (DQ573780 and DQ587821) had putative LINE‐1 retrotransposon targets. This suggests that while some of these survival‐related piRNAs may act on transposons directly, most may exert their effects indirectly or through alternative mechanisms.

These findings support a novel model in which piRNAs promote longevity through mechanisms beyond their canonical role in transposon repression. Evidence from model organisms is consistent with this hypothesis. In *C. elegans* global reduction of piRNA levels doubled lifespan (Huang et al. 2023). In *Drosophila melanogaster*, tissue‐specific loss of *piwi* genes produced context‐dependent effects on lifespan that varied by sex, tissue type, and stress state (Proshkina et al. 2021). Loss of *piwi* in neurons of both sexes, in the male fat body, and in irradiated fat body tissue of both sexes increased lifespan, accompanied by activation of stress response genes in these tissues. Our 5‐year human data similarly implicate smRNA‐related stress‐response pathways as contributors to increased survival. Additional evidence from *C. elegans* links systemic RNA interference (RNAi) pathways to lifespan regulation: overexpression of components such as *SID‐1*, *SID‐2*, and *SID‐5* shortened lifespan, likely by disrupting the balance between intracellular and extracellular RNA—a phenomenon termed Intracellular/Extracellular Systemic RNA imbalance (InExS) (Camara et al. 2024). Specifically, intestinal overexpression of *SID‐2* increased uptake of double‐stranded (ds)RNA from the gut and was sufficient to reduce lifespan, suggesting that excessive uptake of dsRNAs (including smRNAs such as miRNAs with hairpin structures) can be detrimental through InExS (Camara et al. 2024). Alternatively, the low circulating levels of smRNAs—particularly piRNAs—associated with greater human survival may reflect increased tissue retention, where their local functions in transposon suppression or stress adaptation could support healthy aging.

Our analysis also identified a distinct set of miRNAs having a role in survival. For example, miR‐153‐3p was positively associated with 5‐year survival, aligning with its known role in cellular stress response pathways (Matai and Slack 2023). These results reflect the importance of miRNAs in proteostasis and cellular response to aging, particularly in hematopoietic stem cell function (Ortiz et al. 2023). Only four smRNAs, all piRNAs, were estimated direct causal determinants of 2‐year survival and were shared predictors of 5‐year survival; there was no overlap of 2‐year or 5‐year survival predictors with 10‐year smRNA predictors. In keeping with the known consistent association of physical activity with lower risk of mortality, particularly in older adults (Martinez‐Gomez et al. 2024), MBs for all time horizons included one or more measures of physical activity. Surprisingly, age was not a predictor variable in the 10‐year survival MBs but given the low predictivity of 10‐year modeling, these results should be interpreted with caution. Taken together with the low prediction provided by the molecular measures of 10‐year survival, the results show the ability of the studied smRNAs to meaningfully predict short‐ and medium‐term survival only.

Wang et al. (2023) profiled miRNAs in B‐cells from Ashkenazi Jewish centenarians and 70‐year‐old controls without a family history of longevity, identifying 49 differentially expressed (DE) miRNAs, most of which were upregulated in centenarians and linked to the insulin/IGF‐1 signaling (IIS) pathway. A total of 43 of these 49 miRNAs were detected in D‐EPESE plasma; however, only miR‐18a‐5p appeared in one MB model as a predictor of 10‐year survival in D‐EPESE and was higher in both long‐lived D‐EPESE individuals and Ashkenazi centenarians. In general, expression patterns for the 43 shared miRNAs in the two cohorts were largely consistent, with regulation occurring in the same direction. This concordance was especially strong for miRNAs predicting 5‐ and 10‐year survival (≥ 86% agreement) but was lower for those linked to 2‐year survival (35% agreement). For 2‐year survival, piRNAs DQ593963, DQ573780, and DQ587821 emerged as stronger predictors of survival as they superseded these miRNAs in sepset analyses, suggesting piRNAs may serve as more direct/non‐confounded indicators. This result aligns with *Drosophila* germline data indicating that the PIWI‐piRNA pathway is upstream of the miRNA pathway by controlling miRNA biogenesis through DICER1 (Lenart et al. 2018).

Currently, a few commercially marketed products aim to quantify biological aging, most of which rely on DNA methylation markers, including Novos Age, MyDNAge, the Index Biological Age test, and the TallyAge test. Additionally, some tests, such as the TruAge Complete test, assess biological aging based on telomere length. However, no available tests to date utilize smRNA measures. Given the regulatory role of smRNAs in DNA methylation and gene expression, exploring the relationship between the hard endpoint of mortality and associated smRNAs and DNA methylation patterns presents a promising avenue for future research. Such analyses could potentially bridge the gap between smRNA function and existing methylation‐based aging clocks, offering a more comprehensive understanding of the molecular determinants of aging.

Strengths of this paper included the use of an integrated predictive and causal approach—MB analysis, combined with sepset analysis and do‐calculus for causal effect estimation—to identify probable direct causes of survival with a multi‐tiered approach to validation. The MB of outcome variables of interest, such as survival, combines both predictive and local causal properties, as it represents the minimal set of variables that collectively provide the maximum information about the outcome (Guyon et al. 2007). Under the distributional assumption of faithfulness and the causal sufficiency condition, there is only one MB and it consists of the direct causes, direct effects, and direct causes of direct effects of the outcome of interest (Aliferis et al. 2010a; Pearl 2009; Spirtes et al. 2001); when the outcome, such as survival/non‐survival, is terminal, the MB contains only the direct causes of the outcome (Statnikov et al. 2013). When faithfulness is violated, multiple statistically indistinguishable MBs may exist (the so‐called *equivalence class* problem), and only one of the many MBs is the *causal* MB. While information equivalence prevents us from identifying the precise causal factor among the putative direct causes of survival, it does not preclude estimation of the effects attributable to the true causal factor. Moreover, in this work, we generated all MBs for each survival horizon, identifying the most probable direct causes of survival as the features consistently present in 100% of models. We also estimated the anticipated benefit to survival with modification of smRNA alone, thereby providing theoretical thresholds for clinical interventions to promote survival. In the first stage of Discovery, a nested cross‐validation protocol within the Discovery Subset portion of the Discovery dataset eliminated bias during model selection and error estimation. Internal validation further confirmed unbiasedness in the Discovery cohort. Another strength of this work included the use of an independent External Validation cohort, processed, sequenced, and analyzed separately from the Discovery cohort. This showed a slight nominal decline in predictive performance between the discovery and independently assayed validation datasets, indicating some sensitivity to RNA sequencing batch effects. However, the impact was not critical, supporting the feasibility of future clinical applications in which the test could be applied to individual patients using their own sequencing data. Together with permutation tests confirming model unbiasedness and statistical significance of the signature, this approach demonstrated model robustness while accounting for potential technical variability in smRNA measurements. Additionally, our study showed that the Qiagen platform specific output required minimal downstream data filtering and manipulation but maintained data quality and predictivity, which supports its potential for direct application at the individual patient level and may facilitate the translation of genomic insights into clinical practice.

While all samples originated from the same population‐based cohort, we believe the double‐holdout design—especially the final, independently processed External Validation sample—meets or exceeds the definition of external validation, functioning as a separate group of individuals, distinct from the data used to build the model, and used to assess the model's performance and generalizability. Although mechanistic wet‐lab studies of piRNA/miRNA function could further illuminate biological pathways, such experiments are beyond the scope of the present study, which is focused on rigorous biomarker discovery and validation using untargeted and unbiased approaches in a large human cohort with biospecimens and clinical data. Notably, three top‐ranked miRNAs identified here have already been experimentally validated to regulate biological processes relevant to aging and tissue repair; all three candidates were higher at baseline in 5‐year survivors compared with deceased participants, consistent with their direction of benefit reported in the literature. For example, miRNA miR‐214‐3p promotes anabolic activity in human articular cartilage from adults with osteoarthritis (Hsueh et al. 2025); miRNA miR‐125b‐5p protects against muscle atrophy and inflammation by targeting TRAF6 (Qiu et al. 2019; Rasheed et al. 2019); and miRNA miR‐365a‐3p counteracts skeletal muscle aging by promoting muscle stem cell proliferation and differentiation, at least in part by reversing the inhibitory effect of its direct target, HOXA9 (Zhu et al. 2021). These findings underscore the biological plausibility of our results and highlight promising avenues for future mechanistic investigation.

Our piRNA target gene list, based on up to one mismatch of piRNAs with targets, should be regarded as preliminary due to the limitations of current interrogation tools and the challenge of accurate prediction, as random mismatches within piRNA sequences do not significantly affect targeting efficiency (Jiang et al. 2024). Consequently, the interactions between piRNAs and their predicted target genes require experimental validation. Further limitations arise from the detection of numerous piRNA isoforms (isopiRs) by small RNA‐sequencing. The functional equivalence of these isopiRs to canonical piRNAs remains unclear and warrants further investigation. Additionally, the Qiagen mapping pipeline used in this project reflects the evolving nature of piRNA research, as considerably less is known about piRNAs compared to miRNAs. Target prediction tools for piRNAs are still under development, highlighting the need for continued refinement and validation of these methodologies.

In contrast to previous studies focusing predominantly on miRNAs, our work broadened the scope to small non‐coding RNAs, revealing a key role for circulating piRNAs in survival, particularly over short (2‐year) and medium‐term (5‐year) periods. Our findings provide compelling evidence that circulating smRNAs—especially piRNAs—are powerful predictors of survival in older adults and potential biomarkers of longevity. The development of a streamlined, high‐accuracy predictive model, along with the identification of specific piRNAs associated with survival, underscores the biological relevance of these molecules in aging. Notably, our results suggest that piRNAs may influence longevity through novel mechanisms beyond their traditional roles in the germline, offering exciting opportunities for therapeutic strategies to promote healthy aging.

## 3 Methods

### 3.1 Statistical Machine Learning and Bioinformatic Analysis

#### 3.1.1 Predictive Modeling

The survival outcome was dichotomous, alive vs. deceased 2‐, 5‐, or 10‐years after the blood draw (the event marking time zero for survival measurement) at year 6, when participants were aged ≥ 71 years. We derived models to predict survival at these three different time horizons using combinations of the 828 smRNAs, 186 clinical variables, and age to assess the information content of these data modules. For the first stage model discovery and independent validation, we aimed to establish internal validity given smRNA data measured within the same assay batch. We divided the Expanded Discovery dataset (*N* = 707, smRNA assessed with one assay batch) into a Discovery Subset (*N* = 505) and Internal Validation (*N* = 202) datasets. We used the Discovery Subset to build predictive models; we conducted model selection (inner loop of the stratified repeated NCV), and performance estimation in the outer loop of the NCV and, subsequently and independently on the Internal Validation dataset. For the second stage of model discovery, we aimed to establish external validity of our models, specifically, the generalizability of our models across different (non‐overlapping) batches of biological assays and samples. We used the Expanded Discovery dataset for model selection and performance estimation via NCV to build the final predictive model (with the best hyper parameters identified). This model was then tested on an independent External Validation dataset (*N* = 564), consisting of separate samples with smRNA sequenced as a separate distinct batch (Figure 1). We examined two model selection schemes, one optimized for predictive performance, and a second that jointly optimized predictive performance and parsimony to facilitate clinical implementation and deployment. The above protocols are statistically unbiased (i.e., exhibit no overfitting of error estimates) both theoretically and as empirically tested by label permutation procedures (Ojala and Garriga 2010); they allow for extensive model selection (hence reducing under‐fitting). The “stacking” of nested cross‐validation and hold‐out validation with regularization of the classifiers employed and strong feature selector algorithms provided four layers of protection against overfitting (Simon and Aliferis 2024).

#### 3.1.2 Markov Boundary (MB) Analysis

To identify predictive variables with likely causal interpretability for survival, we employed MB analysis (see Methods S1 for details) as previously described (Kraus et al. 2022). In brief, a MB of an outcome variable is a non‐reducible variable set that renders all other variables independent of the outcome, therefore contains maximum information regarding the outcome and (in faithful distributions) maximum parsimony (Pearl 2009; Tsamardinos and Aliferis 2003). We used the GLL algorithm to identify the MBs (Aliferis et al. 2010b). According to causal graph theory, MB variables are direct causes (under the assumption of no unmeasured confounding—“causal sufficiency”—and given that the predictive variable is a terminal one). Because multiple MBs can exist in certain distributions, it is useful to identify the full equivalence class of MBs, that is, all possible direct causal candidate sets. To achieve this, we extracted all MBs for each time horizon using our previously published, theoretically sound, and extensively benchmarked TIE* algorithm (Statnikov et al. 2013), and then estimated ranges of their direct causal effect estimates using Pearl's do‐calculus, which guarantees unbiased and complete causal effect estimation. The effect estimation was computed by fitting a logistic regression model using the variables in each MB variable set as the independent variables and the corresponding outcome as the dependent variable. In addition, we applied a Sepset analysis (Spirtes et al. 2001), extended as described in Kraus et al. (2022), to explain why some previously reported mortality risk factors were rejected from our final models and to shed light on the information content of clinical variables compared to smRNAs.

#### 3.1.3 Causal Interpretation of Markov Boundary Analysis

Our analysis identifies variables within the intersection of all equivalent Markov Boundaries (MBs) as those most plausibly representing true causal factors. Under causal sufficiency, all variables in this intersection can be interpreted as causal; when causal sufficiency is violated, some may be non‐causal but remain statistically indispensable for prediction. Variables outside the intersection of equivalent MBs have a different interpretation. Under causal sufficiency, this set contains a mixture of causal and non‐causal variables; without causal sufficiency, some variables in this set may again be non‐causal. Importantly, no true measured causes are excluded by this procedure. When violations of causal sufficiency raise the possibility of false causal positives, only a relatively small number of targeted experiments are needed to distinguish true causal variables from non‐causal correlates, compared with the full original variable set.

## Author Contributions

V.B.K., S.M., C.‐H.C., and C.F.A. conceived the study. C.‐H.C., J.L.H., and C.G.V. performed wet laboratory experiments. S.M., S.S., M.C.O., C.G.V., and C.F.A. curated the data. S.M., S.I.N., and C.F.A. performed the formal statistical analyses. X.Z. and C.G.V. performed network analyses. V.B.K., W.E.K., H.J.C., and C.F.A. acquired the funding for this study. H.J.C. conducted the clinical investigations for this study. V.B.K., S.M., S.I.N., W.E.K., M.C.O., X.Z., C.G.V., S.S., J.L.H., C.‐H.C., E.K., and C.F.A. devised the methodologies used for this study. V.B.K., S.M., and C.F.A. were responsible for all project administration. S.M. and C.F.A. were responsible for the computing resources, software, supervision, and data validation. V.B.K. and C.F.A. were responsible for the overall project supervision. V.B.K., S.M., S.I.N., X.Z., C.G.V., E.K., and C.F.A. produced the graphics for visualization of the results. V.B.K., S.M., S.I.N., and C.F.A. drafted the manuscript. All authors confirm that they had full access to all the data in the study and accept responsibility for submitting it for publication. V.B.K., S.M., S.I.N., and C.F.A. had direct access and verify the underlying data reported in the manuscript. All authors read and approved the final version of the manuscript.

## Funding

The study was supported by funding from NIH/NIA R01AG054840 (to V.B.K., S.M., S.I.N., W.E.K., H.J.C., M.C.O., C.G.V., J.L.H., and C.F.A.), the Duke Claude D. Pepper Older Americans Independence Center NIH/NIA P30‐AG028716 (to V.B.K., J.L.H., W.E.K., and H.J.C.), NIH/NCATS UL1TR002494 (to S.M. and C.F.A.), NIH U54AG076041 (to C.F.A.), and NIH/NHLBI 1UM1TR004405 (C.F.A.).

## Ethics Statement

Annual approval was provided by Duke University Institutional Review Board (approval number Pro00010226).

## Consent

Written informed consent was provided by all participants.

## Conflicts of Interest

Constantin F. Aliferis is co‐inventor in several patents on machine learning modeling methods but receives no income from them. The other authors declare no conflicts of interest.

## Supporting information


**Data S1:** Supplementary Methods.
**Figure S1:** Markov Boundary (MB) smRNAs selected through smRNA only analyses.
**Figure S2:** Targets of longevity‐associated piRNAs.
**Figure S3:** STRING network analysis of the mRNA targets of the miRNAs identified in 5‐year longevity–predictive models.

**Table S1:** Predictive performance of models built and validated using the Expanded Discovery dataset (*N* = 707).
**Table S2:** Performance of models built with smRNA_PSO vs. smRNA_TMM with and without other variables.
**Table S3:** Descriptive statistics of variables identified as Markov Boundary (MB) variables.
**Table S4:** Models and their predictive performance optimizing for predictive performance or predictive performance and parsimony.
**Table S5:** Causal modeling. Characteristics of estimated Markov Boundaries (MBs) and their predictive performances on the independent External Validation dataset (*n* = 564).
**Table S6a:** Causal analysis. Tie coefficients for 2‐year survival with their standardized effect estimates in each Markov Boundary (MB) model; all smRNA are platform specific output (PSO) values. TOP PANEL: Markov Boundary smRNA and clinical features selected through analyses of all features. BOTTOM PANEL: Markov Boundary smRNA selected through smRNA only analyses.
**Table S6b:** Causal analysis. Tie coefficients for 5‐year survival with their standardized effect estimates in each Markov Boundary (MB) model; all smRNA are platform specific output (PSO) values. TOP PANEL: Markov Boundary smRNA and clinical features selected through analyses of all features. BOTTOM PANEL: Markov Boundary smRNA selected through smRNA only analyses.
**Table S6c:** Causal analysis. Tie coefficients for 10‐year survival with their standardized effect estimates in each Markov Boundary (MB) model; all smRNA are platform specific output (PSO) values. TOP PANEL: Markov Boundary smRNA and clinical features selected through analyses of all features. BOTTOM PANEL: Markov Boundary smRNA selected through smRNA only analyses.
**Table S7:** Estimated change in survival probability for hypothetical survival promoting intervention on estimated candidate causes.
**Table S8:** Sepset analysis of all variables (smRNA_PSO, clinical variables, and age) for all time horizons.
**Table S9:** Predicted targets of longevity smRNA for all time horizons.
**Table S10:** Significant pathways identified via functional enrichment analysis of mRNAs predicted by survival‐associated smRNAs.

## Data Availability

EPESE data are publicly available through the National Archive of Computerized Data on Aging (NACDA) at https://doi.org/10.3886/ICPSR09915.v3. Additional deidentified participant data related to the D‐EPESE cohort are available upon request from the corresponding author under a data sharing agreement. Processed sequencing data are deposited in the Synapse repository and available for download without restriction using Project SynID syn65643335. Computer code: Codes pertaining to methods in this paper are previously published and are also available upon request from the corresponding author under a material sharing agreement.
