# Supplementary material for: Select Small Non‐Coding RNAs Are Determinants of Survival in Older Adults
Source: Aging Cell. 2026 Feb 24;25(3):e70403. doi: 10.1111/acel.70403 (PMC12933132; doi:10.1111/acel.70403)
Supplement: Supplementary file 1 — Data S1: Supplementary Methods. Figure S1: Markov Boundary (MB) smRNAs selected through smRNA only analyses. Figure S2: Targets of longevity‐associated piRNAs. Figure S3: STRING network analysis of the mRNA targets of the miRNAs identified in 5‐year longevity–predictive models. [file ACEL-25-e70403-s001.docx]

**SUPPLEMENTARY METHODS & FIGURES for**

**Select Small Non-coding RNAs are Determinants of Survival in Older Adults**

Virginia Byers Kraus, Sisi Ma, Syeda Iffat Naz, Xin Zhang, Christopher G Vann, Melissa C Orenduff, William E Kraus, Steven Shen, Janet L Huebner, Ching-Heng Chou, Erich Kummerfeld, Harvey Jay Cohen, Constantin F Aliferis. ^^^equal contributors

**SUPPLEMENTARY METHODS**

**Inclusion and Ethics statement.** Written informed consent was provided by all participants. Subsequent annual approval was provided by Duke University Institutional Review Board (approval number Pro00010226). Samples for the D-EPESE cohort were collected under this ongoing IRB oversight with additional approval and informed contain obtained from all participants specifically for the year 6 blood draw in 1992 (Kraus et al., 2022).

**D-EPESE sample and sample selection.** As previously described (Kraus et al., 2022), D-EPESE was a 10-year longitudinal study of a stratified random sample of 4,162 community-based residents ≥65 years of age, living in five adjacent counties in the piedmont area of North Carolina. Using the Kish procedure (Kish, 1965), only one person 65 years of age or older was selected from a chosen household. The sample was designed to consist of at least 50% Black older adults. As previously described (Cornoni-Huntley et al., 1990), area sampling was used at the first stage of the design to obtain a sample of 1980 census blocks, block clusters, and enumeration districts. The second stage of sampling was the selection of smaller geographic areas, or segments, within the primary units. A listing unit was designated in each sampling area, and the listing of the included housing units was compiled. Each housing unit was categorized by observation as Black or non-Black. The third stage sample consisted of approximately 26,183 of the pre-listed housing units. Racial composition of the sample was controlled by stratification of the pre-listed sampling units according to the race categorization. Screening interviews were attempted at all sample housing units for identification of persons 65 years of age or older. The fourth stage of the sampling was the random selection of one person 65 years of age or older within each household containing such persons. Of the eligible sample members selected, 54% of participants were Black. All in-person (P) interviews, including those at baseline (P1 in 1986/87), 3 years (P2 in 1989/90), and 6 years (P3 in 1992/93) after baseline, were performed in the study participants’ homes. All other interviews were performed using computer-assisted telephone interviewing (CATI) by interviewers who telephoned sample members at their homes. At P3, of those interviewed (2,569 survivors), 67% (1,727) provided consent for blood sampling; 60% (1,554) had a successful in-home, non-fasting blood draw with sufficient volumes for storage for future use. Among other characteristics, those not having blood drawn were either unable to give consent, generally because of cognitive dysfunction (269), or refused to have blood drawn, were unavailable, or technically could not be drawn (573). There were significant differences (p<0.05) among the three groups, i.e. blood drawn, unable to consent, and refusers, respectively as follows, (means): age 71.6, 77.0, 72.5 years; % female 65.0, 72.1, 78.0; % Black 52.3, 61.7, 57.8; Katz ADL 0.3, 3.1, 0.5; Rosow-Breslau 1.0, 2.6, 1.3; Nagi 1.8, 1.3, 2.0; life satisfaction 10.6, n/a, 17.8; self-rated health 2.4, n/a, 2.9; and % cognitively impaired by SPMSQ 12.4, 78.8, 15.0. There were no differences in % urban (53.7, 52.4, 57.4), years of education (9.0, 7.0, 8.8), or % depressed (8.8, n/a, 10·2). EPESE study protocol data are publicly available through the National Archive of Computerized Data on Aging (NACDA) at https://doi.org/10.3886/ICPSR09915.v3. Death data were obtained from National Death Index (NDI) searches through December 31, 2019.

To provide separate Discovery and Validation datasets for this study, the D-EPESE cohort with plasma biospecimens {Cornoni-Huntley, 1990 #13384} was randomly divided into two approximately equal subsets (n=727 and n=728) with stratification to ensure balance by race, sex, age, and survival status. During the discovery process, the N=707 Expanded Discovery data were further randomly divided into Discovery Subset (N=505) and Internal Validation (N=202) datasets for model construction, model selection, and independent validation. For the second stage, aimed at validating the models and assessing their generalizability, we used an independent External Validation subset of participants from the remaining half of the D-EPESE cohort; sequencing was performed on a cost-constrained sample enriched for participants who died within two years, yielding a final analytic sample of n=564. The size of this independent External Validation dataset was determined through a power analysis, informed by results obtained from the Discovery analyses, and the available project funding for sequencing additional samples. The independent External Validation dataset included all 102 remaining individuals who died within two years of blood acquisition, and 462 individuals randomly chosen from the remaining 605. This set had a slightly higher rate of death from all causes than the full population but was otherwise representative. Because our measure of predictivity was AUC, an evaluation metric that is independent of prior probability, our model validation using this metric was not biased by discrepancies in the all-cause death rate. The plasma of the Expanded Discovery and independent External Validation subsets were sequenced for smRNA in separate batches, resulting in a total sequenced sample of n=1,271.

**Small RNA extraction and sequencing.** Small RNA from plasma (200 µl) was isolated using the Qiagen miRNeasy Serum/Plasma Advanced Isolation Kit (Catalog no. 217204), with MS2 carrier RNA at a final concentration of 0.8 µg/µl, in accordance with the manufacturer’s specifications and stored at -80°C until analysis. RNA was suspended in 20 μL RNAse-free water and underwent quantification via the Qubit^™^ RNA HS Assay Kit (Thermo Fisher Scientific; Waltham, MA, USA) with 17 µl submitted to the Duke University Center for Genomic and Computational Biology for library preparation (Qiagen QIAseq miRNA Library kit Cat ID: 331505) and sequencing on the Illumina NovaSeq6000 (Illumina; San Diego, CA, USA). All samples underwent 76 base pair single-end sequencing resulting in a final depth of 21.3M reads/sample yielding 716 miRs and 173 piRs in the Expanded Discovery cohort, and 30.3M reads/sample yielding 720 miRs and 177 piRs in the External Validation cohort.

**Processing and normalization of small RNA sequencing data.** Small RNA sequencing data were preprocessed using the Qiagen GeneGlobe miRNA Primary Quantification Server – Legacy 2.0 pipeline. Briefly, raw reads were aligned to the Hg38 reference genome with nested sequences being collapsed into a single readout. Sequence adapters and low-quality bases were then removed using CutAdapt(Martin, 2011). The unique molecular identifier (UMI)-tools package (Smith, Heger, & Sudbery, 2017) was used to identify insert sequences and UMIs; reads with <16bp for insert sequences (i.e. reads that were too short) and <10bp for UMI sequences (defective UMIs) were discarded. Bowtie2 was used to map miRNA sequences to miRBase (v21) (Kozomara & Griffiths-Jones, 2014) while piRNA sequences were mapped to piRNABank (Sai Lakshmi & Agrawal, 2008). Finally, FeatureCounts (Liao, Smyth, & Shi, 2014) was used to quantify genomic features prior to statistical analysis. Statistical analysis of small RNA compared survival prediction based on Qiagen platform-specific output (PSO) counts vs counts normalized via the trimmed mean of M-values (TMM) method as we previously reported (Vann et al., 2022) with normalized values scaled to counts per million (cpm).

**Identification of mRNA targets of MB identified survival smRNA.** We performed smRNA-to-mRNA target prediction for smRNAs identified through MB equivalence class modeling. Potential piRNA-to-mRNA interactions were identified via sequence complementarity using piRNAQuest (v2.0), piRBase (v3.0), piRNAdb (v1.8), and via NCBI BLAST using the reverse complement sequence, allowing for a single nucleotide mismatch for each piRNA-to-mRNA pairing (Chirn et al., 2015; Ghosh et al., 2022; Wang et al., 2019). We also used RepeatMasker to investigate associations with identified piRNA and genomic repeat elements (Smit, 2013). MiRNA-to-mRNA target identification was completed using miRTarBase (v9.0) (Huang et al., 2022), requiring each predicted target to have at least one publication verifying the union using well accepted methods (i.e., reporter assay, western blot, RT-qPCR, microarray/sequencing), and NCBI BLAST queries with reverse complement sequences (Ghosh et al., 2022).

**STRING analysis.** The Search Tool for the Retrieval of Interacting Genes/Proteins (STRING) (Szklarczyk et al., 2019) was used to identify targets of miRNAs. For STRING analyses we used the ‘proteins only’ query, with a minimum required interaction score of 0.4 indicating medium confidence. The active interaction sources included co-expression, co-occurrence, databases, experiments, gene fusion, neighborhood, and text mining. We identified mRNAs targeted by the 18 miRNAs associated with 5-year survival prediction. To investigate the functional relationships among their shared targets, we conducted a network analysis using the STRING database (v11.5). This analysis identified enriched Reactome pathways and biological processes, with statistical significance determined by a false discovery rate (FDR) threshold of <0.05.

**Statistical and Bioinformatic Analysis**

***Predictive Modeling****:*

Overall Analytic design: Our overall goal was to: (1) test the predictive capability of smRNA along with other data modalities for survival, and (2) construct and assess the capability for deployment in the clinical setting. The protocols were statistically unbiased (i.e., exhibited no overfitting of error estimates) both theoretically and empirically as demonstrated by the label permutation procedures (Ojala & Garriga, 2010); they allowed for extensive model selection (hence reducing under-fitting). The ‘stacking” of nested cross-validation and hold-out validation with regularization of the classifiers employed and feature selector algorithms, provided four layers of protection against overfitting. Data analysis was conducted using Matlab 2022 and R 4.4.1.

Model Development: We used three feature selection methods: all features, support vector machine recursive feature elimination, and GLL [generalized local learning, which infers MBs of the outcome (Aliferis, Statnikov, Tsamardinos, Mani, & Koutsoukos, 2010a, 2010b)], and four classifiers: logistic regression, random forests, Boosted Trees, and support vector machines, chosen because of their theoretical properties and excellent empirical performance across many biomedical domains/datasets and especially clinical and omics biomolecular data in relatively low sample size domains (Chowdhury et al., 2022; Christodoulou et al., 2019; G. Simon & Aliferis, 2024; A. Statnikov, Aliferis, Tsamardinos, Hardin, & Levy, 2005; A. Statnikov, Wang, & Aliferis, 2008; Sufriyana et al., 2020). The hyper-parameter for the GLL was K= [1,2,3]. The hyper-parameter for random forest was 500 or 1000 trees, mtry=sqrt (number of variables). The hyper-parameter for boosted tree was 500 or 1000 trees, learning rate of 0.1, using a tree stump. For support vector machines, we used the Cartesian product of the following hyper parameters: polynomial kernel degree= [1,2,3] and C=[0.1,1,10]. These resulted in 70 combinations of feature selection, classification method and hyper-parameters. Missing data were handled by mean imputation, and we included indicator variables for missingness. The imputation was done according to the training data at all stages of the model development and performance estimation to prevent information leakage between train-test splits leading to overfitting.

Model Selection: To select the features, classification method and hyper-parameter combinations for predicting survival, we used stratified repeated nested cross-validation (NCV, four repeats and five folds) to achieve optimal model selection, avoid overfitting, and obtain unbiased performance estimations. The determination of the optimal combination of feature selection methods, model types and hyperparameter combinations was conducted in the inner loop of the NCV. We considered two model selection schemata, which lead to two sets of models: (1) to select the model optimizing predictive performance, the model selection procedure selects among all 70 combinations of feature selection, classification method and hyper-parameters based on best average predictive performance in the NCV inner-loop; (2) to select the model optimizing predictive performance and parsimony, the model selection procedure selects among the 42 combinations of the GLL feature selection methods, all classification method, and hyper-parameters. Focusing the model selection among the models with the GLL feature selection results in models with smaller number of predictors and thus produces models that are more cost effective for implementation and deployment.

Performance Estimation and Validation: The performance estimation of the selected model on the Discovery Subset or the Expanded Discovery data was conducted in the outer loop of the NCV and in the Internal or External Validation data respectively (Duda, Hart, & Stork, 2000; A Statnikov, 2011). Area under the receiver operating characteristic curve (AUC) was the metric for performance estimation; AUC ranges from 0 to 1, with AUC of 1 indicating perfect prediction and AUC of 0.5 indicating prediction at a chance level. We note that this procedure is very conservative in that it stacks two separate unbiased estimators to eliminate any possibility for inflated predictivity estimates. It can be interpreted as a fully independent sample validation of a previously fully-cross-validated optimal model (R. Simon, Radmacher, Dobbin, & McShane, 2003). Model performance comparison was performed using student’s t-test (2 tailed) for nested cross validation results and Delong’s test for holdout validation (i.e. internal validation and external validation) results (DeLong, DeLong, & Clarke-Pearson, 1988).

***Markov Boundary (MB) Analysis*:**

A MB (A. Statnikov, Lytkin, Lemeire, & Aliferis, 2013) of an outcome variable is by definition a minimal (i.e., non-reducible) variable set that renders all other variables independent of the outcome. It contains all the information available in the dataset regarding the outcome. Removing a variable set from a MB results in loss of information regarding the outcome, whereas adding any other variable set (from the observed distribution) does not increase the information. Multiple MBs (and associated optimal predictive models) can exist in certain distributions and indeed in our data, when examining smRNA, clinical data and age together, we identified 7 MBs for predicting survival at 2-years, 115 MBs for 5-years, and 10 MBs for 10-years. When examining smRNA data alone, we identified 3 MBs for predicting survival at 2-years, 18 MBs for 5-years, and 3 MBs for 10-years. These are the three equivalence classes of optimal predictor sets and corresponding local causal models corresponding to the three studied time horizons for all data and smRNA data respectively. Each MB model for a particular time horizon contains equivalent information to other models in the same equivalence class. One of the multiple MBs contains precisely and only the local direct causes of survival of a particular time horizon. Multiple MBs may contain a subset of the local direct causes. However, every MB is statistically indistinguishable from the other MBs. The estimated effects of each MB variable controlling for the rest are accurate if the outcome is fully determined by the variables, or if no local confounder is involved; otherwise, the estimated causal effect is an upper bound on the true effect under the assumption of monotonic causal influences (i.e., effect of confounder and of measured local cause do not cancel partially). These observations hold within each MB member of the equivalence class. Equivalent variable sets across equivalent MBs are interchangeable statistically, have the same estimated causal effects and can only be resolved by experimentation or by prior knowledge. Bivariate or multivariate highly collinear variables are a special case of information equivalence and represent a tiny fraction of the equivalence classes. Because of these considerations it is essential for robust discovery that analyses do not fail to consider all statistically indistinguishable possibilities, i.e., selecting an arbitrary member of the equivalence class (as is common practice), will generate many false positives and false negatives). In the present study we safeguard against this possibility by deriving the full MB equivalence class for each time horizon using specialized algorithms.

Discovering Markov Boundaries Equivalence Class: Intuitively, equivalent MBs or Markov Boundary equivalence classes can stem from the presence of overlapping or redundant information in candidate predictors of the outcome of interest. In other words, they are target information equivalent (TIE) (A. Statnikov et al., 2013). The potential existence of equivalent MBs due to TIE distributions is an instance of an *equivalence class* problem. Equivalence model classes present problems for discovery insofar as multiple models share optimal data fit. If the analyst (as is common practice) uses methods that discover one member of the class and ignores the rest, the larger is the equivalence model class, and the smaller the probability that an arbitrarily chosen optimally-fitting model is causal. In our approach, we overcome this pitfall by using algorithms that discover *all* optimal models in the equivalence class and then dissect the contents of the member models looking for variables in *all* equivalent models (that are guaranteed to be causal under causal sufficiency), and variables that are interchangeable (which may or may not be causal but they are clearly marked as such). Finally, we note that for estimating causal effects, if variables A and B are information equivalent to Outcome Ox, they will have the same estimated causal effect. In other words, one can reliably estimate the true causal effect even without knowing with certainty which specific variable—among several equivalent ones—is actually causal. Therefore, for each outcome of interest (2-year survival, 5-year survival, 10-year survival), we applied the TIE* algorithm to derive all MBs of that outcome. The following parameters were used for TIE*: Fisher’s test as the conditional independence test, alpha=0.05 as the level of significance, max-k of 3, max-card of 100, and the independence criterion for deciding the validity of a newly discovered MB.

TIE* uses MB induction subroutines tailored to the distribution at hand to identify the set of maximally predictive and non-redundant signatures for that data distribution. TIE* has mathematically proven correctness and completeness properties and due to its use of MB procedures, is very resistant to over fitting in high dimensional data. In empirical tests, in contrast to other methods lacking such properties, TIE* generates complete predictor model equivalence classes (“signatures”) that are perfectly reproducible in independent datasets. TIE* also is computationally most efficient in the sense that it never generates any non-MB set, thus ensuring scalability (limited only by the size of the equivalence class). Moreover, the application of the TIE* algorithm to various datasets, general as well as biomedical, has resulted in collections of highly accurate and predictively equivalent risk models (Karstoft, Galatzer-Levy, Statnikov, Li, & Shalev, 2015; Ma et al., 2020; A. Statnikov & Aliferis, 2010). Thus, TIE* has excellent empirical sample and computational efficiency; in extensive empirical studies, TIE* not only extracts many more maximally predictive and non-redundant signatures than all previous methods, but TIE* signatures are also reproducible in independent datasets whereas signatures produced by previous methods are often not reproducible or have lower predictivity (A. Statnikov & Aliferis, 2010). TIE* is guaranteed to be correct in the large sample under its stated assumptions. In the case of a small sample, some signatures that are not maximally predictive and/or redundant will be statistically indistinguishable from the maximally predictive and non-redundant ones. A more technical observation is that the equivalence classes under study comprise both sampling variance-induced indistinguishability (present in the small sample and vanishing in the large sample), as well as structural information equivalence (that exists in the small sample and persists in the large sample). Our analytic approach based on the TIE* algorithm family captures both kinds of equivalence/multiplicity.

Causal Effect Estimation in presence of equivalent Markov Boundaries (MBs): In the presence of equivalent MBs, and no latent, only one MB is the exact set of the direct causes, direct effects, and direct cause of direct effects. True local causes however can appear in many (and in fact all) MBs in the equivalence class (the precise % is determined by the structure of the causal process that generates the data). The equivalent MBs are statistically indistinguishable from one another, and the true direct causes within these equivalent MBs cannot be identified statistically without prior knowledge and/or experimentation. Therefore, since they are all potential causes of the target of interest, we provide a range of potential causal effect estimations for all variables in the equivalent MBs. The effect estimation was computed by fitting a logistic regression model using the variables in each MB variable set as the independent variables and the corresponding outcome as the dependent variable. Pearl’s do-calculus guarantees that in the absence of unmeasured confounders, this direct causal effect estimation procedure is unbiased (Pearl, 2009).

Sepset Analysis: Any variable that is not part of any MB for the outcome of interest does not provide any information regarding the target of interest given one or more of the variables in the MB (Spirtes, Glymour, & Scheines, 2001). Several risk/protective factors for survival identified in prior literature are not part of any MB in our study. The sepset analysis as conducted here elucidates which variables in a MB block influence/subsume the information in a particular variable (e.g. previously identified risk/protective factors of survival) that is not part of the MB. Specifically, by conducting conditional independence testing where the conditioning sets are all subsets of the union of all MBs, we derived a sepset for each variable that was not part of any MB. For brevity’s sake, we report only one sepset for each variable even though multiple sepsets may exist. This novel local sepset procedure (LSL, for local sepset learning) has the following properties. (a) The explanatory power of sepsets as justification for dropping variables from consideration as local causes is restricted to the space of possible local causes and not delegated to confusing and inconsistent remote sepsets. Moreover, (b) by applying GLL on the union of variables in the MB equivalence class we can obtain these localized sepsets very efficiently (i.e., no need to examine all subsets of that union in a brute force manner). Finally, (c) because the equivalence variable sets in the MB equivalence class are readily obtained by TIE*, we can generate the equivalence class of all local sepsets for some variable(s) of interest. We call this extension of LSL: Local Sepset Learning with Information Equivalences (LSLIE).

**References**

Aliferis, C., Statnikov, A., Tsamardinos, I., Mani, S., & Koutsoukos, X. (2010a). Local causal and Markov blanket induction for causal discovery and reature selection for classification Part I: Algorithms and empirical evaluation. *J of Machine Learning Research, 11*, 171-234.

Aliferis, C., Statnikov, A., Tsamardinos, I., Mani, S., & Koutsoukos, X. (2010b). Local causal and Markov blanket induction for causal discovery and reature selection for classification Part II: Algorithms and empirical evaluation. *J of Machine Learning Research, 11*, 235-284.

Chirn, G. W., Rahman, R., Sytnikova, Y. A., Matts, J. A., Zeng, M., Gerlach, D., . . . Lau, N. C. (2015). Conserved piRNA Expression from a Distinct Set of piRNA Cluster Loci in Eutherian Mammals. *PLoS Genet, 11*(11), e1005652. doi:10.1371/journal.pgen.1005652

Chowdhury, M. Z. I., Naeem, I., Quan, H., Leung, A. A., Sikdar, K. C., O'Beirne, M., & Turin, T. C. (2022). Prediction of hypertension using traditional regression and machine learning models: A systematic review and meta-analysis. *PLoS One, 17*(4), e0266334. doi:10.1371/journal.pone.0266334

Christodoulou, E., Ma, J., Collins, G. S., Steyerberg, E. W., Verbakel, J. Y., & Van Calster, B. (2019). A systematic review shows no performance benefit of machine learning over logistic regression for clinical prediction models. *J Clin Epidemiol, 110*, 12-22. doi:10.1016/j.jclinepi.2019.02.004

Cornoni-Huntley, J., Blazer, D., Lafferty, M., Everett, D., Brock, D., & Farmer, M. (1990). Established Populations for Epidemiologic Studies of the Elderly. In *Resource Data Book* (Vol. Vol II). Washington DC: PHS, NIH.

DeLong, E., DeLong, D., & Clarke-Pearson, D. (1988). Comparing the areas under two or more correlated receiver operating characteristic curves: a nonparametric approach. *Biometrics, 44*, 837–845.

Duda, R., Hart, P., & Stork, D. (2000). *Pattern Classification* (2nd ed.). Hoboken: John Wiley & Sons.

Ghosh, B., Sarkar, A., Mondal, S., Bhattacharya, N., Khatua, S., & Ghosh, Z. (2022). piRNAQuest V.2: an updated resource for searching through the piRNAome of multiple species. *RNA Biol, 19*(1), 12-25. doi:10.1080/15476286.2021.2010960

Huang, H. Y., Lin, Y. C., Cui, S., Huang, Y., Tang, Y., Xu, J., . . . Huang, H. D. (2022). miRTarBase update 2022: an informative resource for experimentally validated miRNA-target interactions. *Nucleic Acids Res, 50*(D1), D222-D230. doi:10.1093/nar/gkab1079

Karstoft, K. I., Galatzer-Levy, I. R., Statnikov, A., Li, Z., & Shalev, A. Y. (2015). Bridging a translational gap: using machine learning to improve the prediction of PTSD. *BMC Psychiatry, 15*, 30. doi:10.1186/s12888-015-0399-8

Kish, L. (1965). *Survey Sampling*. New York: John Wiley & Sons.

Kozomara, A., & Griffiths-Jones, S. (2014). miRBase: annotating high confidence microRNAs using deep sequencing data. *Nucleic Acids Res, 42*(Database issue), D68-73. doi:10.1093/nar/gkt1181

Kraus, V. B., Ma, S., Tourani, R., Fillenbaum, G. G., Burchett, B. M., Parker, D. C., . . . Aliferis, C. F. (2022). Causal analysis identifies small HDL particles and physical activity as key determinants of longevity of older adults. *EBioMedicine, 85*, 104292. doi:10.1016/j.ebiom.2022.104292

Liao, Y., Smyth, G. K., & Shi, W. (2014). featureCounts: an efficient general purpose program for assigning sequence reads to genomic features. *Bioinformatics, 30*(7), 923-930. doi:10.1093/bioinformatics/btt656

Ma, S., Schreiner, P. J., Seaquist, E. R., Ugurbil, M., Zmora, R., & Chow, L. S. (2020). Multiple predictively equivalent risk models for handling missing data at time of prediction: With an application in severe hypoglycemia risk prediction for type 2 diabetes. *J Biomed Inform, 103*, 103379. doi:10.1016/j.jbi.2020.103379

Martin, M. (2011). Cutadapt removes adapter sequences from high-throughput sequencing reads. *2011, 17*(1), 3. doi:10.14806/ej.17.1.200

Ojala, M., & Garriga, G. (2010). Permutation tests for studying classifier performance. *J Machine Learning Research, 11*, 1833-1863.

Pearl, J. (2009). *Causality, Models, Reasoning, and Inference* (second ed.).

Sai Lakshmi, S., & Agrawal, S. (2008). piRNABank: a web resource on classified and clustered Piwi-interacting RNAs. *Nucleic Acids Res, 36*(Database issue), D173-177. doi:10.1093/nar/gkm696

Simon, G., & Aliferis, C. (2024). An Appraisal and Operating Characteristics of Major ML Methods Applicable in Healthcare and Health Science. In G. J. Simon & C. Aliferis (Eds.), *Artificial Intelligence and Machine Learning in Health Care and Medical Sciences: Best Practices and Pitfalls* (pp. 95-195). Cham: Springer International Publishing.

Simon, R., Radmacher, M. D., Dobbin, K., & McShane, L. M. (2003). Pitfalls in the use of DNA microarray data for diagnostic and prognostic classification. *J Natl Cancer Inst, 95*(1), 14-18. doi:10.1093/jnci/95.1.14

Smit, A. H., R; Green, P. (2013). RepeatMasker Open-4.0. Retrieved from <www.repeatmasker.org>

Smith, T., Heger, A., & Sudbery, I. (2017). UMI-tools: modeling sequencing errors in Unique Molecular Identifiers to improve quantification accuracy. *Genome Res, 27*(3), 491-499. doi:10.1101/gr.209601.116

Spirtes, P., Glymour, C., & Scheines, R. (2001). *Causation, Prediction, and Search* (Second ed.): MIT Press.

Statnikov, A. (2011). *A gentle introduction to support vector machines in biomedicine: Theory and methods*. Singapore: World Scientific Pub.

Statnikov, A., & Aliferis, C. F. (2010). Analysis and computational dissection of molecular signature multiplicity. *PLoS Comput Biol, 6*(5), e1000790. doi:10.1371/journal.pcbi.1000790

Statnikov, A., Aliferis, C. F., Tsamardinos, I., Hardin, D., & Levy, S. (2005). A comprehensive evaluation of multicategory classification methods for microarray gene expression cancer diagnosis. *Bioinformatics, 21*(5), 631-643. doi:10.1093/bioinformatics/bti033

Statnikov, A., Lytkin, N. I., Lemeire, J., & Aliferis, C. F. (2013). Algorithms for discovery of multiple Markov boundaries. *J Machine Learning Research, 14*, 499-566.

Statnikov, A., Wang, L., & Aliferis, C. F. (2008). A comprehensive comparison of random forests and support vector machines for microarray-based cancer classification. *BMC Bioinformatics, 9*, 319. doi:10.1186/1471-2105-9-319

Sufriyana, H., Husnayain, A., Chen, Y. L., Kuo, C. Y., Singh, O., Yeh, T. Y., . . . Su, E. C. (2020). Comparison of Multivariable Logistic Regression and Other Machine Learning Algorithms for Prognostic Prediction Studies in Pregnancy Care: Systematic Review and Meta-Analysis. *JMIR Med Inform, 8*(11), e16503. doi:10.2196/16503

Szklarczyk, D., Gable, A. L., Lyon, D., Junge, A., Wyder, S., Huerta-Cepas, J., . . . Mering, C. V. (2019). STRING v11: protein-protein association networks with increased coverage, supporting functional discovery in genome-wide experimental datasets. *Nucleic Acids Res, 47*(D1), D607-d613. doi:10.1093/nar/gky1131

Vann, C. G., Zhang, X., Khodabukus, A., Orenduff, M. C., Chen, Y. H., Corcoran, D. L., . . . Kraus, V. B. (2022). Differential microRNA profiles of intramuscular and secreted extracellular vesicles in human tissue-engineered muscle. *Front Physiol, 13*, 937899. doi:10.3389/fphys.2022.937899

Wang, J., Zhang, P., Lu, Y., Li, Y., Zheng, Y., Kan, Y., . . . He, S. (2019). piRBase: a comprehensive database of piRNA sequences. *Nucleic Acids Res, 47*(D1), D175-D180. doi:10.1093/nar/gky1043

**SUPPLEMENTARY FIGURES**

*
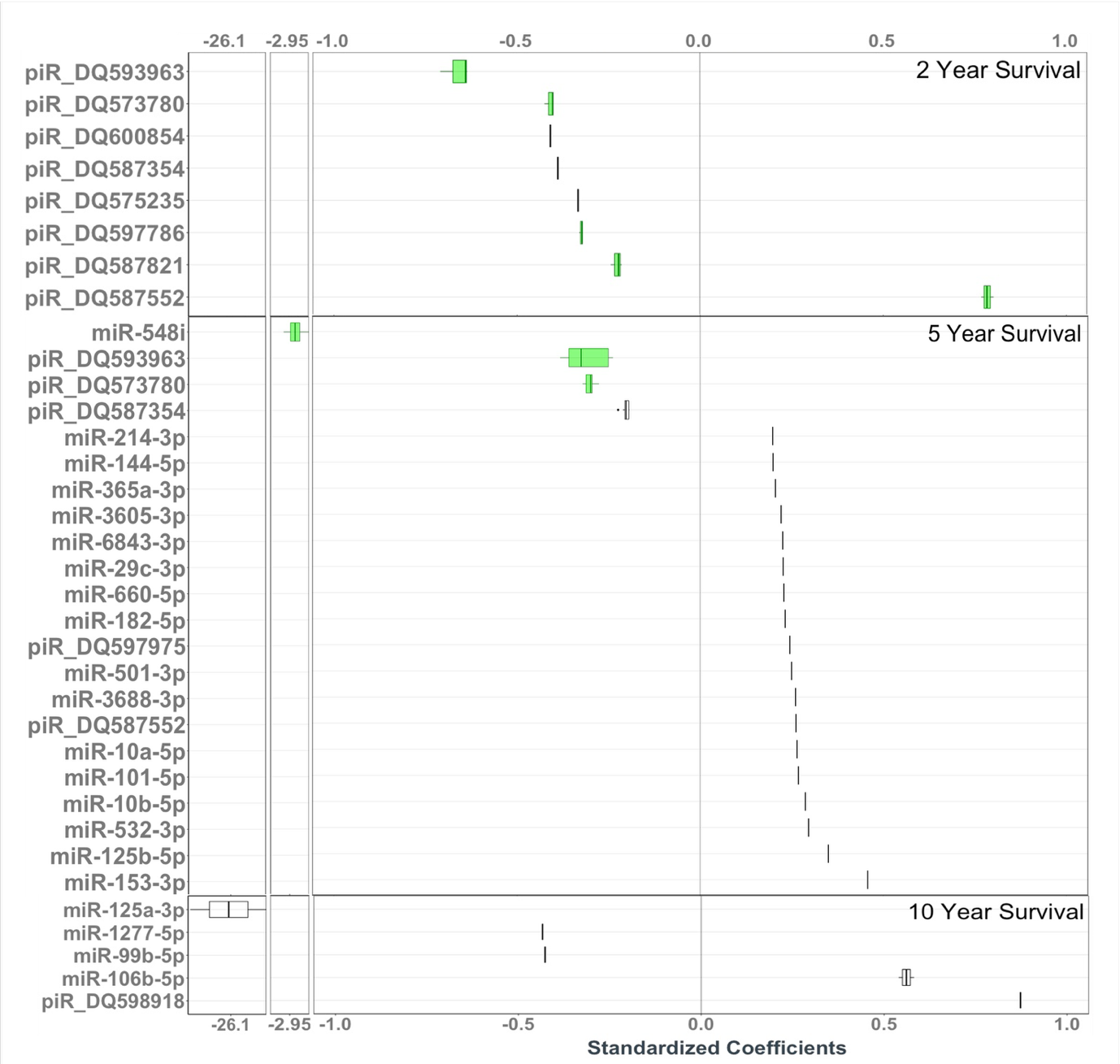
*

**Figure S1**. **Markov Boundary (MB) smRNAs selected through smRNA only analyses.**


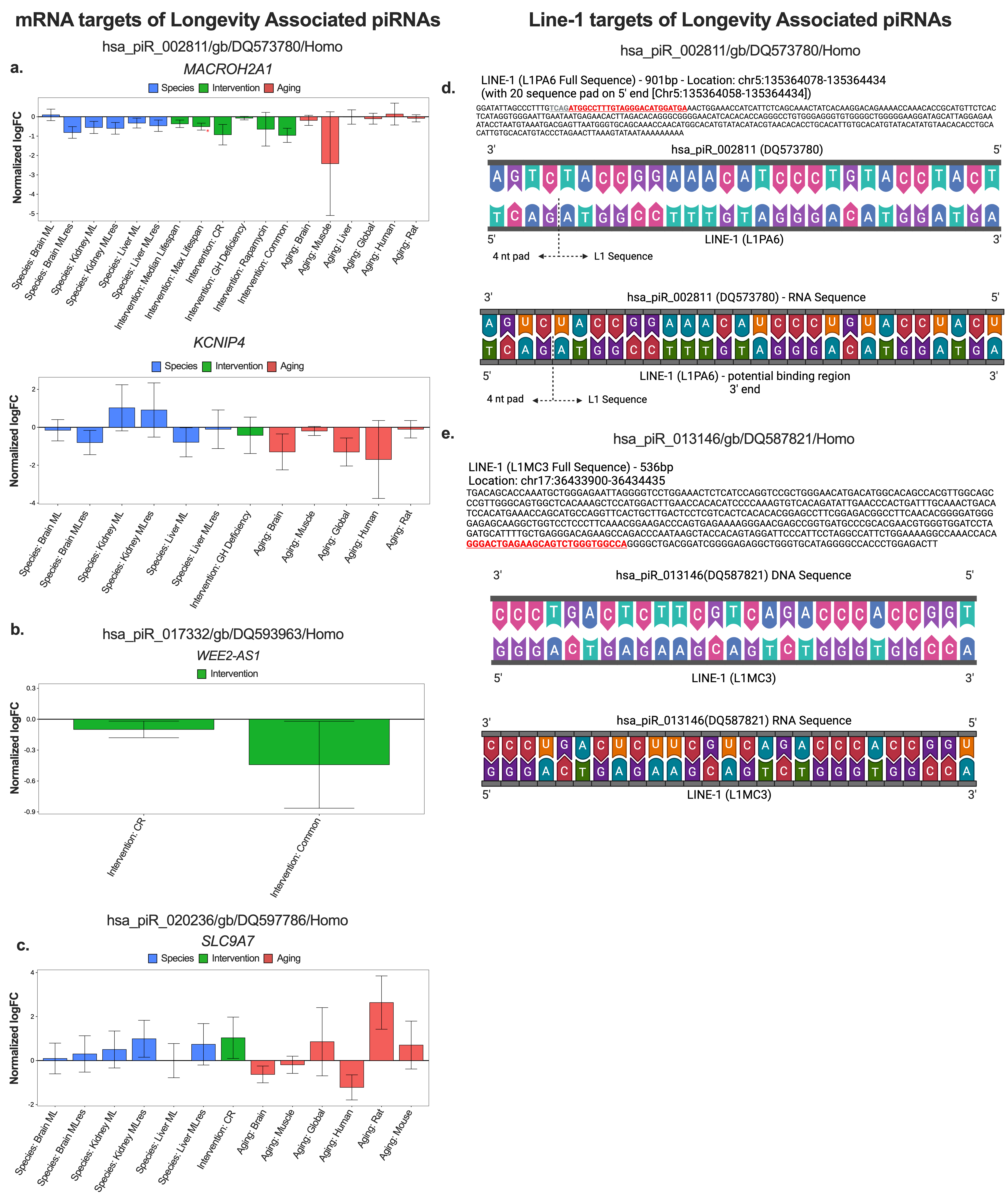


**Figure S2**. **Targets of survival-associated piRNAs. Panels A-C:** messenger RNA (mRNA) targets of survival-associated piRNAs identified in our investigation, were queried using the mSALT database (http://gladyshevlab.org/mSALT/). Data are presented as bar plots depicting summary data generated in mSALT for three piRNAs for which putative mRNA targets could be generated (as described and listed in **Table S9**) using (piRQuest/piRBase/piRNAdb or NCBI BLAST searches for complementary sequences with up to 1nt mismatch: **A**) hsa_piR_002811/gb/DQ573780/Homo_pso, **B**) hsa_piR_017332/gb/DQ593963/Homo_pso, and **C**) hsa_piR_020236/gb/DQ597786/Homo_pso. mRNA expression (y-axes) is presented as normalized log fold change showing the known associations between gene expression and lifespan. Although putative mRNA targets (*TBC1D3F* and *TBC1D3B*) were predicted for hsa_piR_013146/gb/DQ587821/Homo_pso, no data on these mRNA targets were available in mSALT. Additionally, has_piR_002811/gb/DQ573780/Homo_pso was predicted to target OR2AP1 which was not identified in the mSALT database. **D-E)** Line-1 targets of survival associated piRNAs were identified using RepeatMasker (https://repeatmasker.org). Data are presented as sequence complementarity between: **D**) Line-1 PA6 (L1PA6) and hsa_piR_002811/gb/DQ573780/Homo_pso and, **E**) Line-1 MC3 (L1MC3) and hsa_piR_013146/gb/DQ587821/Homo_pso. Abbreviations: ML, Maximum Lifespan; MLres, Maximum Lifespan adjusted for body mass; logFC, log fold change; CR, caloric restriction; GH, growth hormone. Description of summary signatures. Species: Brain ML, Kidney ML, Liver ML, the association of maximum lifespan of mammalian species and gene expression in the brain, kidney, and liver, respectively. Brain MLres, Kidney MLres, and Liver MLres make the same associations between maximum lifespan and gene expression while adjusting for adult body weight. Interventions: median and maximum lifespan represent the association between the effect size of lifespan extending intervention on mouse median and maximum lifespan, and gene expression in liver. Interventions: CR, GH deficiency, and rapamycin represent the effect of CR, GH deficiency, and rapamycin on gene expression in mouse liver (includes Ames dwarf, Snell dwarf, Laron, and Little mice). ‘Interventions: common’ represents the effect of various longevity interventions in mice on liver gene expression. Aging: brain, muscle, and liver represent the association of gene expression in these tissues with aging across humans, mice, and rats. Aging: human, mouse, and rat represent the association of gene expression in these species with aging across 17 various tissues (multi-tissue signatures). Aging: global represents the association of gene expression across humans, mice, and rats across 17 various tissues (multi-species, multi-tissue signature). * Represents Q-value (Benjamini-Hochberg corrected p-value) <0.1.

**
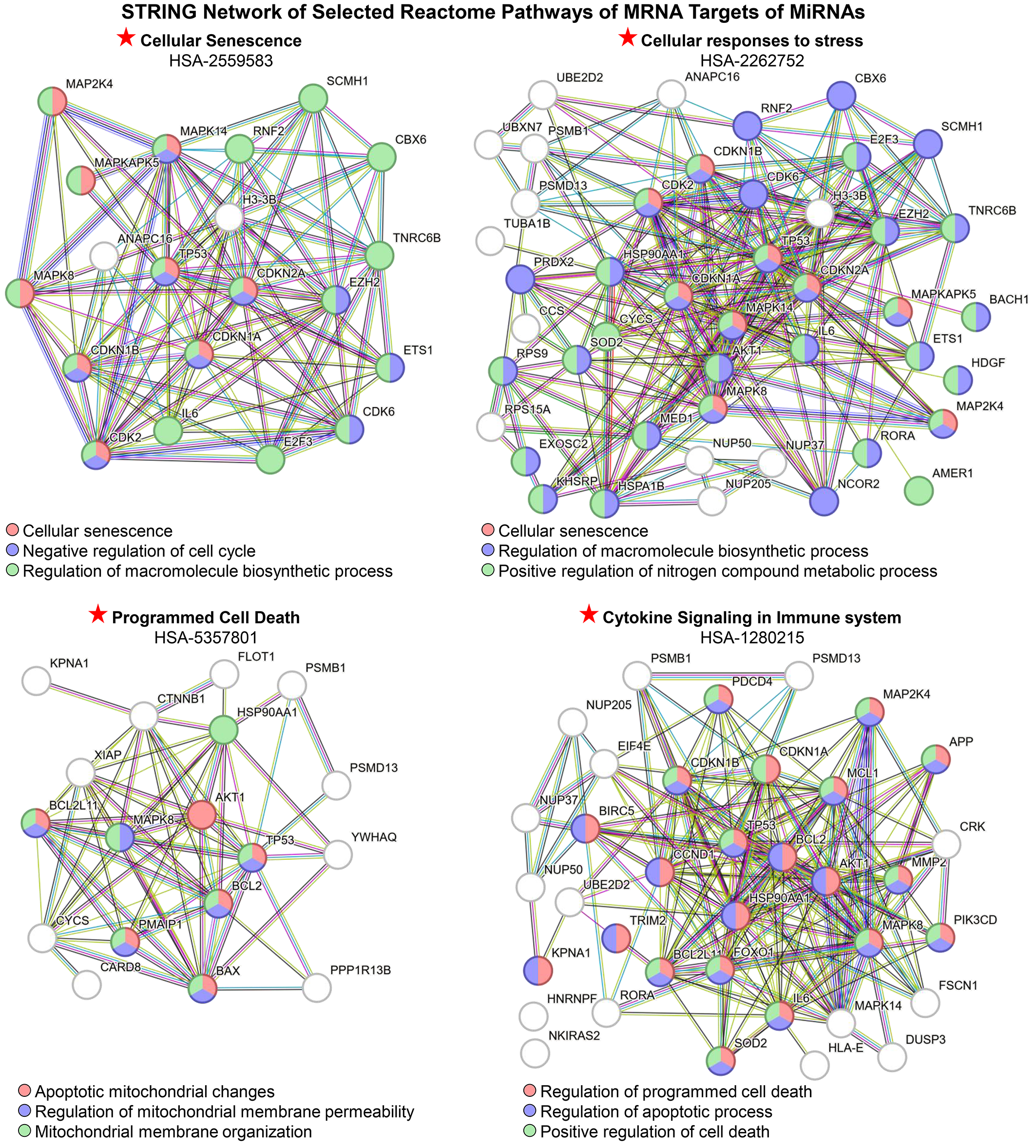
Figure S3.** **STRING network analysis of the mRNA targets of the miRNAs identified in 5-year survival–predictive models.** The STRING graphic depicts networks of mRNAs comprising four selected human Reactome pathways related to senescence (HSA-2559583), apoptosis (HSA-5357801), stress (HSA-2262752) and immune system (HSA-1280215). The colors of each node indicate their involvement in the top 3 enriched biological processes listed in the legend below each graphic. The edges illustrate evidence-based protein associations: light blue and hot pink edges for the known interactions from curated databases and experimental evidence, respectively; green, red and blue edges for the predicted interactions of gene neighborhood, fusions and co-occurrence, respectively; black edges for co-expression; purple edges for protein homology; and yellow edges for text mining.
